# Multi-level omics analysis in a murine model of dystrophin loss and therapeutic restoration

**DOI:** 10.1093/hmg/ddv381

**Published:** 2015-09-18

**Authors:** Thomas C. Roberts, Henrik J. Johansson, Graham McClorey, Caroline Godfrey, K. Emelie M. Blomberg, Thibault Coursindel, Michael J. Gait, C.I. Edvard Smith, Janne Lehtiö, Samir EL Andaloussi, Matthew J.A. Wood

**Affiliations:** 1 Department of Physiology, Anatomy and Genetics, University of Oxford, South Parks Road, OxfordOX1 3QX, UK,; 2 Sanford Burnham Prebys Medical Discovery Institute, Development, Aging and Regeneration Program, 10901 N. Torrey Pines Road, La Jolla, CA92037, USA,; 3 Department of Oncology/Pathology, Cancer Proteomics Mass Spectrometry, SciLifeLab Stockholm, Karolinska Institutet, StockholmSE-171 21, Sweden,; 4 Department of Laboratory Medicine, Karolinska Institutet, HuddingeSE-141 86, Sweden and; 5 Laboratory of Molecular Biology, Medical Research Council, Francis Crick Avenue, CambridgeCB2 0QH, UK

## Abstract

Duchenne muscular dystrophy (DMD) is a classical monogenic disorder, a model disease for genomic studies and a priority candidate for regenerative medicine and gene therapy. Although the genetic cause of DMD is well known, the molecular pathogenesis of disease and the response to therapy are incompletely understood. Here, we describe analyses of protein, mRNA and microRNA expression in the tibialis anterior of the *mdx* mouse model of DMD. Notably, 3272 proteins were quantifiable and 525 identified as differentially expressed in *mdx* muscle (*P* < 0.01). Therapeutic restoration of dystrophin by exon skipping induced widespread shifts in protein and mRNA expression towards wild-type expression levels, whereas the miRNome was largely unaffected. Comparison analyses between datasets showed that protein and mRNA ratios were only weakly correlated (*r* = 0.405), and identified a multitude of differentially affected cellular pathways, upstream regulators and predicted miRNA–target interactions. This study provides fundamental new insights into gene expression and regulation in dystrophic muscle.

## Introduction

The classical monogenic disease Duchenne muscular dystrophy (DMD) is a progressive muscle wasting disorder and the most prevalent muscular dystrophy affecting children. DMD is caused by loss of function mutations in the *DMD* gene, which encodes the dystrophin protein (1,2). Dystrophin is a sub-sarcolemmal structural and signalling protein that acts as an organizing centre for the dystrophin-associated protein complex (DAPC) (3), which serves as a mechanical link between the extracellular matrix and the actin cytoskeleton (4). Loss of dystrophin sensitises muscle fibres to contractile damage (5), leading to chronic cycles of myofibre degeneration and regeneration.

The *DMD* gene consists of 79 exons, many of which code for redundant structural domains (6). DMD is thus amenable to antisense oligonucleotide-mediated splice correction therapy whereby the selective exclusion of one or more exons results in restoration of the translation reading frame. First generation exon skipping therapies show promise in clinical trials (7–11) and second generation peptide-phosphorodiamidate morpholino oligonucleotide (P-PMO) conjugates induce high levels of exon skipping and dystrophin protein restoration in dystrophic *mdx* mice (12–15). Dystrophy in the *mdx* mouse is caused by a nonsense mutation in *Dmd* exon 23 (16,17).

The analysis of gene expression in the *mdx* mouse has the potential to identify (i) novel genes involved in disease pathophysiology, (ii) potential therapeutic targets and (iii) candidate disease biomarkers relevant to DMD. With the rapid development of DNA microarray technology and next-generation sequencing methodologies, analysis of the transcriptome is now commonplace. In contrast, proteomic analysis is substantially more difficult given the increase in biochemical complexity when considering proteins as opposed to nucleic acids. Fibrous tissues, such as muscle, are especially difficult to analyse by mass spectrometry due to high levels of actin and myosin which mask the signals generated by less abundant proteins (18). Similarly, some methodologies, such as two-dimensional gel electrophoresis, are limited only to highly expressed and soluble proteins (19,20). To simplify complex proteomes, we have developed a method based on high resolution isoelectric focusing (HiRIEF) of peptides before nano-LC-MS/MS (liquid chromatography-tandem mass spectrometry) which previously enabled deep proteome coverage in both human and mouse cells (21,22). A number of studies have investigated the proteome in *mdx* mice (summarized in Supplementary Material, Table S1) although these have typically sampled only a small fraction of the proteome due to technical limitations.

Although gene expression studies can be highly informative, their biological interpretation is subject to several limitations. For example, the majority of transcriptomic studies utilizing microarray or RNA-seq methodologies assume that changes in mRNA abundance are matched by corresponding alterations in protein expression, which is often not the case (23–29). As a result, multi-level analyses which simultaneously investigate the proteome and transcriptome have greater potential for providing an understanding of gene regulation and cellular metabolism that might not be possible with any single level of analysis (30). Of particular interest are a class of small RNAs, the microRNAs (miRNAs), which act as regulators of gene expression by binding to target sequences within the 3′ untranslated regions of mRNAs to modulate transcript stability and translation efficiency (31). The importance of miRNAs in shaping the transcriptome and proteome has been widely recognized (32–35), and we have previously investigated differential miRNA expression in the *mdx* mouse (36). However, relatively little is known about miRNA function in dystrophic muscle. miRNA prediction algorithms typically return many hundreds or thousands of predicted targets, and are blind as to whether the miRNA and target mRNA are expressed in the same cell, if at all (37,38). There is therefore a need for empirical validation of mRNA–target interactions due to the high false-positive rates of target prediction algorithms (39,40). The combination of miRNA and mRNA/protein expression data is one method of addressing this problem (41).

In this study, we have used HiRIEF–LC-MS/MS proteomics to profile protein expression in wild-type, *mdx* and P-PMO-treated *mdx* mice with high resolution. In parallel, we have performed mRNA and microRNA arrays in order to provide an integrated proteomic-transcriptomic-miRNomic description of gene expression in the *mdx* mouse.

## Results

### Proteomics analysis of dystrophic muscle

**Figure 1.**
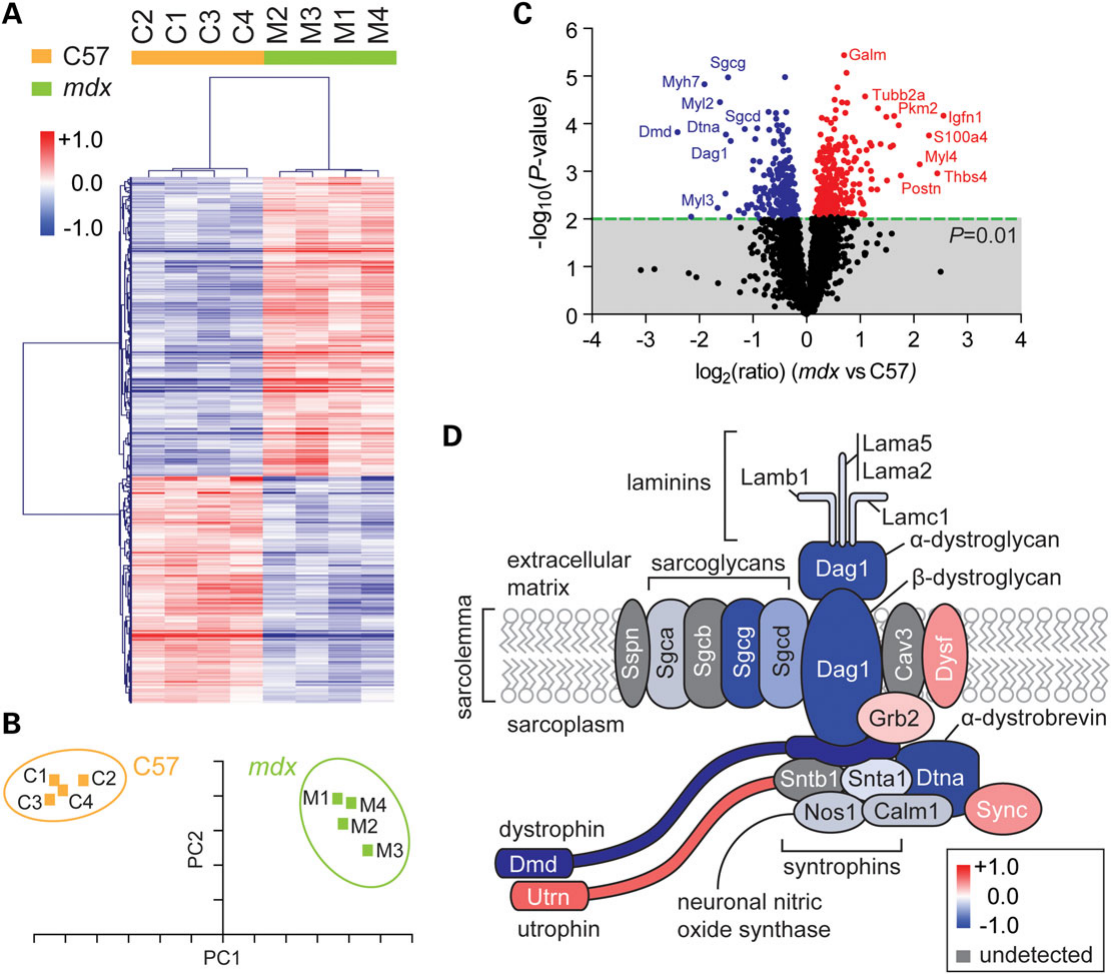
Analysis of the dystrophic proteome. Protein lysates prepared from the TA muscles of 8-week-old C57 and *mdx* mice (*n* = 4) were analysed by LC-MS/MS. Significantly different (*P* < 0.01, *q* < 0.05, *t*-test) proteins were analysed by (**A**) unsupervised hierarchical clustering and (**B**) PCA (the first two components represent 90.9% of the data). (**C**) Identification of differentially expressed genes by volcano plot. (**D**) Schematic of the DAPC. Dystrophin, dystrobrevin, dystroglycans, sarcoglycans, laminins, syntrophin and neuronal nitric oxide synthase are significantly down-regulated. Red indicates up-regulated proteins and blue indicates down-regulated proteins. Grey indicates undetected proteins. Scale bars represent row *Z*-scores.

To investigate the proteome in dystrophic muscle, we performed LC-MS/MS and quantification by iTRAQ (isobaric Tags for Relative and Absolute Quantification). Protein was extracted from serial sections of the tibialis anterior (TA) muscle harvested from 8-week-old male *mdx* mice (*n* = 4) and C57 wild-type controls (*n* = 4). A total of 2869 proteins were identified, of which 2648 (92%) were quantifiable (Supplementary Material, Fig. S1). A Peptide Spectrum Match (PSM) cumulative frequency plot shows that a subset of only 14 highly abundant proteins (e.g. actins, myosins and titin) make up 50% of the protein mass measured (Supplementary Material, Fig. S2). Five hundred and twenty-five proteins (20%) were significantly changed (*P* < 0.01, *q* < 0.05) between *mdx* and C57 (298 up-regulated and 227 down-regulated). Experimental groups formed clearly differentiated clusters with low inter-replicate variation as determined by both hierarchical clustering (Fig. 1A) and Principal Component Analysis (PCA) (Fig. 1A) of statistically significant expression ratios. Similar results were observed using unsupervised analyses (Supplementary Material, Fig. S3). Proteins which exhibited large, highly statistically significant changes in expression were visualized by volcano plot (Fig. 1C). The most down-regulated protein detected was dystrophin (Dmd), which was reduced by ∼81% in the *mdx* samples (*P* = 0.000151) (Supplementary Material, Fig. S4). The muscles of DMD patients and *mdx* mice express low levels of dystrophin on account of endogenous alternative splicing events in a small number of ‘revertant’ myofibres (42,43) and the short isoforms of dystrophin protein are unaffected in *mdx* muscle, meaning that a complete absence of dystrophin protein peptides is not expected. Furthermore, the difference in Dmd expression between C57 and *mdx* muscle is likely an underestimation, as the iTRAQ method suffers from a distortion effect caused by contaminating non-target protein-derived near-isobaric ions which compresses expression ratios towards a value of 1 (44).

Dystrophin was identified based on 108 PSMs, ∼14% protein coverage with 71 unique peptides and 38 PSMs used for quantification, indicating high confidence in dystrophin protein measurement (Supplementary Material, Fig. S1). Notably, the majority of DAPC components were significantly down-regulated, including dystroglycans, sarcoglycans, dystrobrevins, syntrophins, laminins, neuronal nitric oxide synthase and calmodulin-1 (Fig. 1D).

### Partial restoration of the dystrophic proteome by exon skipping therapy

**Figure 2.**
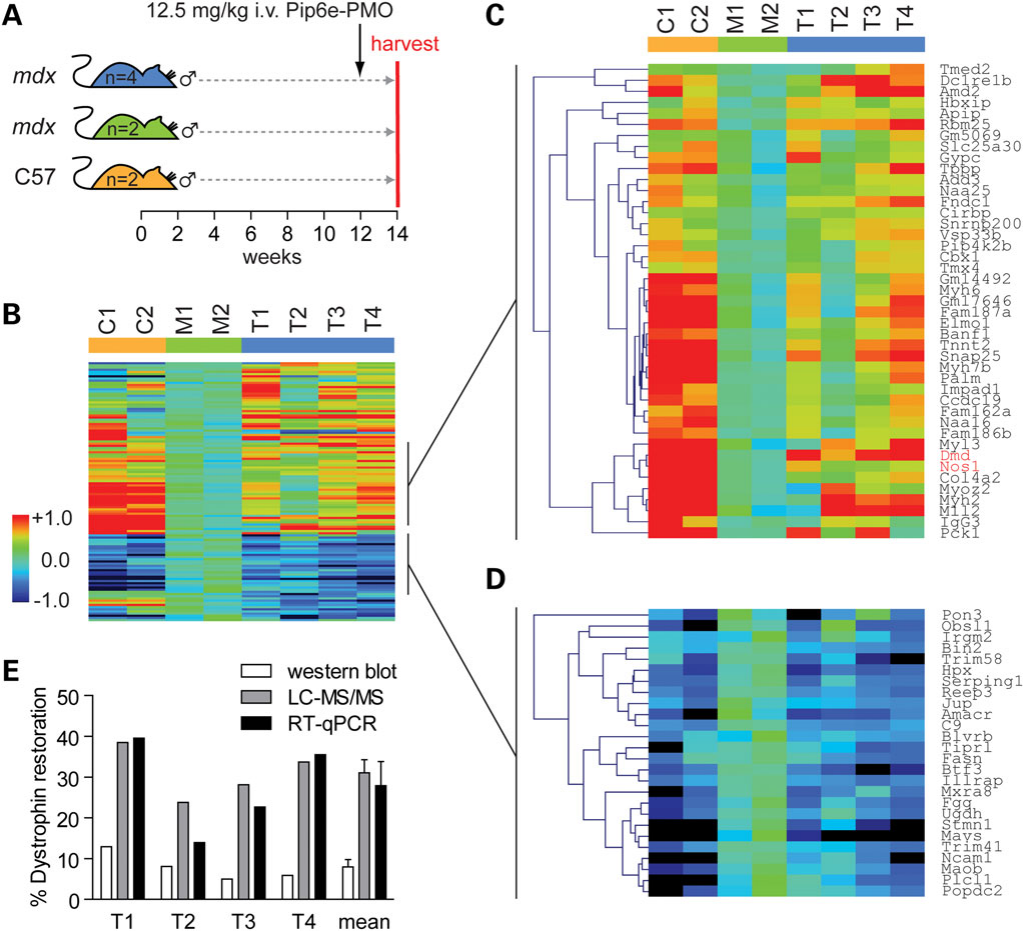
Restoration of the dystrophic proteome by antisense oligonucleotide-mediated exon skipping therapy. (**A**) 12-week-old *mdx* mice (*n* = 4) were injected with a single 12.5 mg/kg intravenous dose of Pip6e-PMO and harvested 2 weeks later. Age-matched (14-week old) C57 and *mdx* controls (*n* = 2) were harvested in parallel. Protein was harvested from TA muscle sections and analysed by LC-MS/MS. (**B**) Heatmap showing protein-clustered protein expression data. Protein expression data were filtered based on inter-replicate variation, leaving 119 proteins. Treatment with Pip6e-PMO resulted in a shift in the dystrophic proteome towards a wild-type-like expression profile. Two clusters show clear proteomic restoration. (**C**) Restored proteins that were down-regulated in *mdx* muscle. Dmd and the DAPC component Nos1 are highlighted in red. (**D**) Restored proteins that were up-regulated in *mdx* muscle. Red and blue indicate up- and down-regulated protein expression, respectively. Green and light blue indicate intermediate protein expression levels. The scale bar represents the row *Z*-score. (**E**) Determination of therapeutic efficacy in individual-treated samples by western blot, LC-MS/MS and RT-qPCR. Values are mean + SEM.

To investigate the response of the dystrophic proteome to exon skipping, we performed a second proteomics experiment comparing 14-week-old C57 (*n* = 2), *mdx* (*n* = 2) and *mdx* mice treated with a single intravenous 12.5 mg/kg dose of a P-PMO conjugate (Pip6e-PMO) (*n* = 4) to restore dystrophin protein expression (Fig. 2A). This time point was selected in order to model muscle in which dystrophic pathology is already established. A total of 3057 proteins were identified, of which 2819 (92%) were quantified. Dystrophin was again detected with high confidence based on 129 PSMs, 10% sequence coverage with 57 unique peptides and 60 PSMs used for quantification (Supplementary Material, Fig. S5) and was the most down-regulated protein in *mdx* muscle (Supplementary Material, Fig. S6).

Multiplexing was limited to eight samples per experiment, and so filtering expression ratios based on statistical significance was not possible due to low *n* numbers. The data were instead filtered by removing proteins with large differences in expression ratio between replicates (Supplementary Materials and Methods), after which 119 proteins remained. Treatment with Pip6e-PMO led to widespread restoration of the dystrophic proteome towards a more wild-type expression profile (Fig. 2B). Although the degree of proteomic restoration following exon skipping therapy was relatively modest, it was commensurate with the magnitude of dystrophin protein rescue. In particular, two clusters of proteins showed a marked response to Pip6e-PMO treatment (Fig. 2C and D). Proteomic restoration was limited in the sample ‘T2’. The therapeutic efficacy of treatment in each individual sample is shown in Figure 2E. Dystrophin protein expression is shown as determined by LC-MS/MS and western blot densitometry, and the percentage of correctly skipped Δexon23 *Dmd* transcripts was assessed by RT–qPCR. RT–qPCR and LC-MS/MS results were tightly correlated (Pearson's *r* = 0.986, *P* = 0.014), and therapeutic efficacy was lowest in sample ‘T2’ as determined by both methods, consistent with the whole proteome LC-MS/MS data. Western blot data were not significantly correlated with the values measured by RT–qPCR or LC-MS/MS (Fig. 2E) although densitometry is less quantitative than the other methodologies.

**Figure 3.**
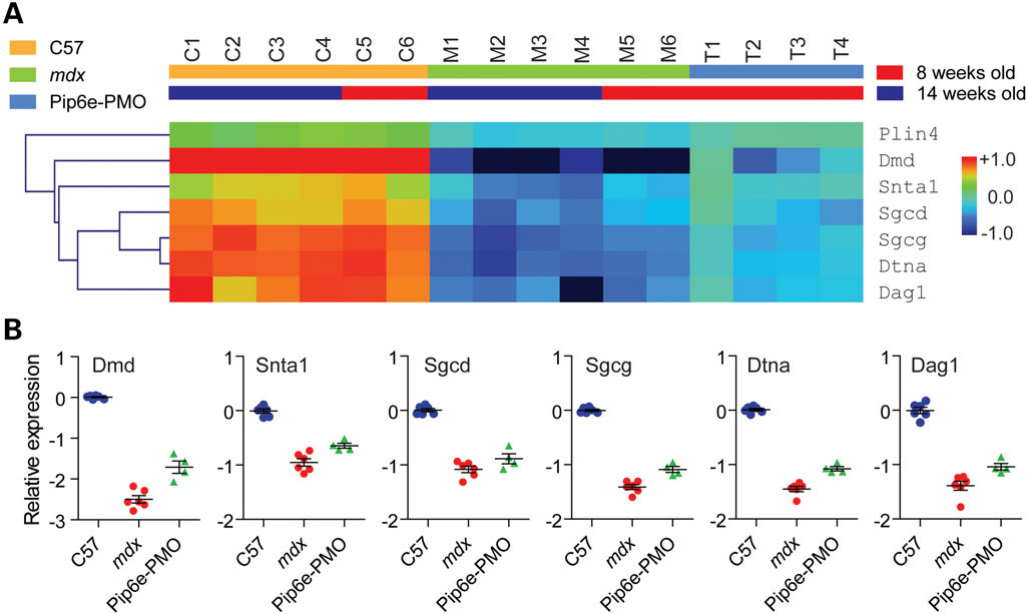
Therapeutic restoration of DAPC components. (**A**) A cluster of significantly differently expressed proteins (labelled † in Supplementary Material, Fig. S7) includes six proteins from the DAPC that shows restoration following therapeutic dystrophin re-expression. Red and blue indicate up- and down-regulated protein expression, respectively. Green indicates an intermediate protein expression level. The scale bar represents row *Z*-scores. (**B**) Plots of individual log_2_ expression ratios for each protein of interest. Values are mean +/− SEM.

The protein expression ratios for 8 and 14 weeks proteomics datasets were positively correlated (Pearson's *r* = 0.681, *P* < 0.0001) (Supplementary Material, Fig. S7A) and 2191 proteins were commonly detected in both datasets (Supplementary Material, Fig. S7B). Given this similarity, expression ratios from both experiments were combined, thereby allowing for statistical analysis by one-way ANOVA. Statistically significant (*P* < 0.01) proteins were sorted by PCA (Supplementary Material, Fig. S7C) and hierarchical clustering (Supplementary Material, Fig. S7D) with samples clustering according to their respective experimental groups. Most notably, a cluster of proteins showed clear therapeutic restoration (labelled † in Supplementary Material, Fig. S7D). Remarkably, this cluster (Fig. 3A) contained Dmd and the DAPC components Snta1, Sgcd, Sgcg, Dtna and Dag1 which were all highly down-regulated in *mdx* muscle and partially restored following treatment with Pip6e-PMO (Fig. 3B). These data show that restoration of dystrophin protein is accompanied by rescue of the expression of DAPC proteins.

### Transcriptomics analysis of dystrophic muscle

**Figure 4.**
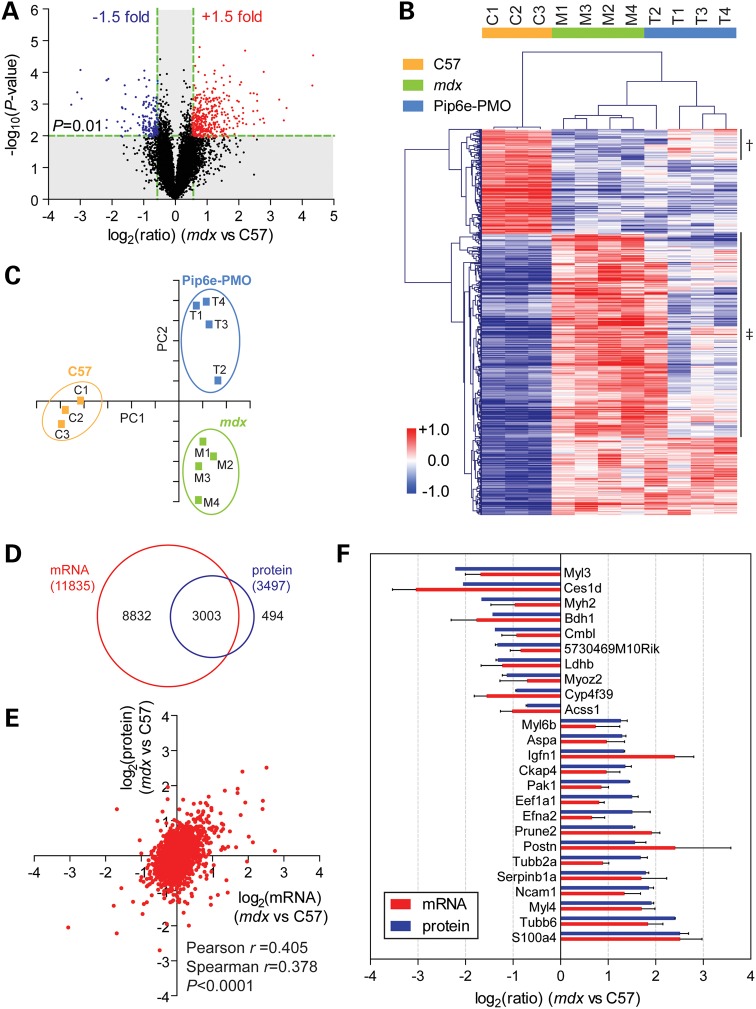
Matched mRNA transcriptomics and proteomics data analysis. RNA extracted from C57, *mdx* and Pip6e-PMO-treated *mdx* TA muscles was analysed by microarray. (**A**) Differentially expressed genes were visualized by volcano plot and filtered so as to include only statistically significantly changed genes (*P* < 0.01, *t*-test) that were changed by more than 1.5-fold in *mdx* muscle. (**B**) Heatmap showing the results of hierarchical clustering analysis of filtered data. Two clusters of mRNAs that were restored towards wild-type levels following treatment with Pip6e-PMO are labelled ‘†’ and ‘‡’, respectively. Red indicates up-regulated proteins and blue indicates down-regulated proteins. The scale bar represents row *Z*-scores. (**C**) PCA of filtered data (the first two components represent 80.5% of the data). (**D**) Venn diagram showing overlap between detected mRNAs and proteins. (**E**) Scatter plot showing the correlation between protein and mRNA expression ratios (2456 mRNA–protein pairs). (**F**) mRNA and protein expression ratios for genes that were concordantly differentially expressed in *mdx* muscle. Genes shown were statistically significant at the *P* < 0.01 level and were differentially expressed by at least 2-fold by either microarray or LC-MS/MS. Values are mean + SEM.

To investigate the relationship between the dystrophic proteome and transcriptome, we performed mRNA microarray analysis on the same tissues used in the 14-week-old proteomics study (additional samples were included to increase *n* numbers for the C57 and *mdx* groups). Resulting mRNA expression ratios were analysed by volcano plot (Fig. 4A), which showed that more mRNAs were up-regulated in *mdx* muscle than were down-regulated. The most differentially expressed mRNAs are shown in Supplementary Material, Figure S8. mRNAs that were significantly (*P* < 0.01) changed by at least 1.5-fold were analysed by hierarchical clustering (Fig. 4B) and PCA (Fig. 4C). Experimental groups were well separated by both analyses, although the treated sample ‘T2’ showed the least restoration and was more closely clustered with *mdx* samples than the other treated samples. Two clusters of mRNAs were restored following treatment (Fig. 4B, labelled † and ‡). These clusters contained 60.5% (302 of 499) of the significantly differentially expressed mRNAs. Genes that were restored towards wild-type levels were enriched for gene ontology terms associated with the extracellular matrix, cell surface, plasma membrane and integrin-mediated signalling, suggesting that specific cellular pathways respond to the restoration of dystrophin (Supplementary Material, Fig. S9A). In contrast, fewer gene ontology terms were enriched (and with lower significance) in the list of non-restored mRNAs (Supplementary Material, Fig. S9B). Of the 11 835 protein-coding genes detected by microarray (defined as those with UniProt identifiers), 3003 (∼25%) were quantified in at least one of the proteomics experiments (Fig. 4D).

Protein and mRNA expression ratios showed a highly significant but weak positive correlation (Pearson's *r* = 0.405, Spearman's *r* = 0.378, both *P* < 0.0001) based on 2456 mRNA–protein pairs (Fig. 4E). Highly differentially expressed genes that were concordantly changed at both protein and mRNA levels are shown in Figure 4F. Notably, DAPC mRNAs were not found to be significantly altered in contrast to the proteomics data (Supplementary Material, Fig. S10).

### Pathway analysis of proteomics and transcriptomics datasets

To extract biological meaning from the proteomics/transcriptomics datasets, *mdx* versus C57 expression ratios from each experiment were analysed using Ingenuity Pathway Analysis (IPA). Multiple canonical pathways were identified as being significantly perturbed in *mdx* muscle (Supplementary Material, Fig. S11). Notably, metabolic pathways were highly perturbed, especially in the 8-week-old *mdx* muscle. Mitochondrial dysfunction was identified as highly significance, with multiple components of the electron transport Complexes I, II, III and V being down-regulated (Supplementary Material, Fig. S12). Pathways linked with the TCA cycle, amino acid degradation, gluconeogenesis and fatty acid metabolism were similarly affected. These findings are consistent with previous reports of metabolic abnormalities in dystrophic muscle (45).

Well-established secondary pathological features of DMD were also identified including those associated with nNOS Signalling in Skeletal Muscle, Calcium Signalling and (Hepatic) Fibrosis. Interestingly, the most significantly dysregulated pathway in the transcriptomics dataset was Axonal Guidance Signalling. Five pathways were commonly differentially regulated in all three omics datasets: Actin Cytoskeleton Signalling, Calcium Signalling, Epithelial Adherens Junction Signalling, ILK Signalling and Tight Junction Signalling (Supplementary Material, Fig. S13).

IPA was used to identify predicted up-stream regulators based on the expression of known down-stream regulated genes (Supplementary Material, Fig. S14). Six up-stream regulators were predicted to be activated or inhibited in both protein and mRNA datasets: Insr, Klf15, Ppargc1a, Prl, Tfam and Tgfb1. Up-regulation of TGFB1 (Transforming Growth Factor-β1) signalling is a known feature of dystrophic muscle (46,47). Tgfb1 is a pro-inflammatory cytokine that coordinates fibrogenesis, in part through suppression of the dystromiR miR-29c (48). Conversely, Klf15 (Krüppel-like Factor 15) signalling was predicted to be down-regulated at both mRNA and protein levels. Klf15 and Tgfb1 act as reciprocal negative regulators of one another. As a result, down-regulation of Klf15 signalling would be expected to have a pro-inflammatory/pro-fibrotic effect via promoting Tgfb1 activation. Equally, Klf15 down-regulation could be a consequence of enhanced Tgfb1 signalling (49).

Conversely, Ppargc1a (Peroxisome proliferator-activated receptor gamma coactivator 1-alpha, PGC1α) signalling was predicted to be down-regulated (signalling by the related proteins Ppara and Pparg was similarly predicted to be down-regulated in the *mdx* muscle proteome). Although PPARGC1A protein was not detected in our study, it has been previously shown to be down-regulated in the vastus lateralis of dystrophic GRMD dogs (50). The primary role of Ppargc1a appears to be the regulation of fatty acid metabolism/storage and glucose utilization, although it also executes a multitude of other functions (51). For example, Ppargc1a regulates mitochondrial biogenesis (52) and so a reduction in its signalling may explain the down-regulation of mitochondrial electron transport proteins observed in dystrophic muscle. Transgenic expression of Ppargc1a in *mdx* muscle reduced dystrophic pathology, underlining the importance of this signalling pathway in dystrophic pathology (53).

Despite the commonalities discussed earlier, substantial differences in pathway dysregulation were observed between the omics datasets underlining the limitations of analyses which focus on a single level of gene expression. However, limited proteome coverage may account for some differentially affected pathways identified at the transcript level but not at the protein level. For example, NF-κB signalling was predicted to be up-regulated (a well described pathological feature of DMD (54)) based on the transcriptomics data only.

### Analysis of the dystrophic miRNome

**Figure 5.**
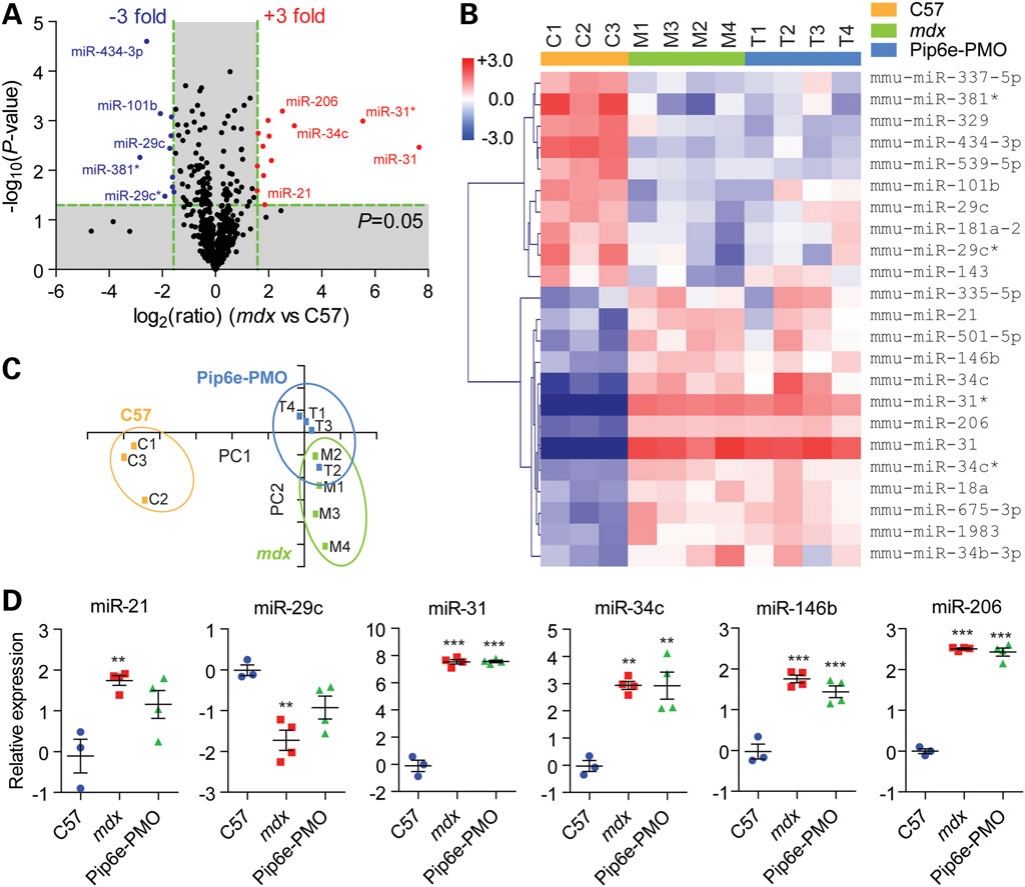
Analysis of the dystrophic microRNA transcriptome. RNA extracted from C57, *mdx* and Pip6e-PMO-treated *mdx* TA muscles was analysed by miRNA microarray. (**A**) Differentially expressed genes were visualized by volcano plot and filtered so as to include only statistically significantly changed genes (*P* < 0.05, *t*-test) that were changed by more than 3-fold in *mdx* muscle. (**B**) Heatmap showing the results of hierarchical clustering (gene-clustered) analysis of filtered data. Red indicates up-regulated miRNAs and blue indicates down-regulated miRNAs. The scale bar represents the row *Z*-score. (**C**) PCA of filtered data (the first two components represent 92.6% of the data). (**D**) miRNA microarray data for specific miRNAs-of-interest. Individual log_2_ expression ratios for each replicate are shown. Mean +/− SEM values are indicated, **P < 0.01, ***P < 0.001, one-way ANOVA and Bonferroni *post hoc* test.

To investigate relationships between miRNA levels and expression of their target genes, we performed miRNA microarray analysis on RNA samples taken from the same mice used in the proteomics and mRNA transcriptomics experiments described earlier. miRNAs that were highly differentially expressed in *mdx* muscle were identified by volcano plot (Fig. 5A). Analysis of these miRNAs by hierarchical clustering (Fig. 5B) and PCA (Fig. 5C) showed that *mdx* and Pip6e-PMO-treated muscles were poorly differentiated, indicating a lack of restoration towards wild-type levels. Focusing on a subset of DMD-associated miRNAs (dystromiRs), miR-21, miR-29c and miR-146b were found to be partially restored towards wild-type expression levels in the treated mice. However, somewhat surprisingly, three of the most highly up-regulated dystromiRs, miR-31, miR-34c and miR-206, showed no significant restoration in response to exon skipping therapy (Fig. 5D).

### miRNA–target interaction networks

**Figure 6.**
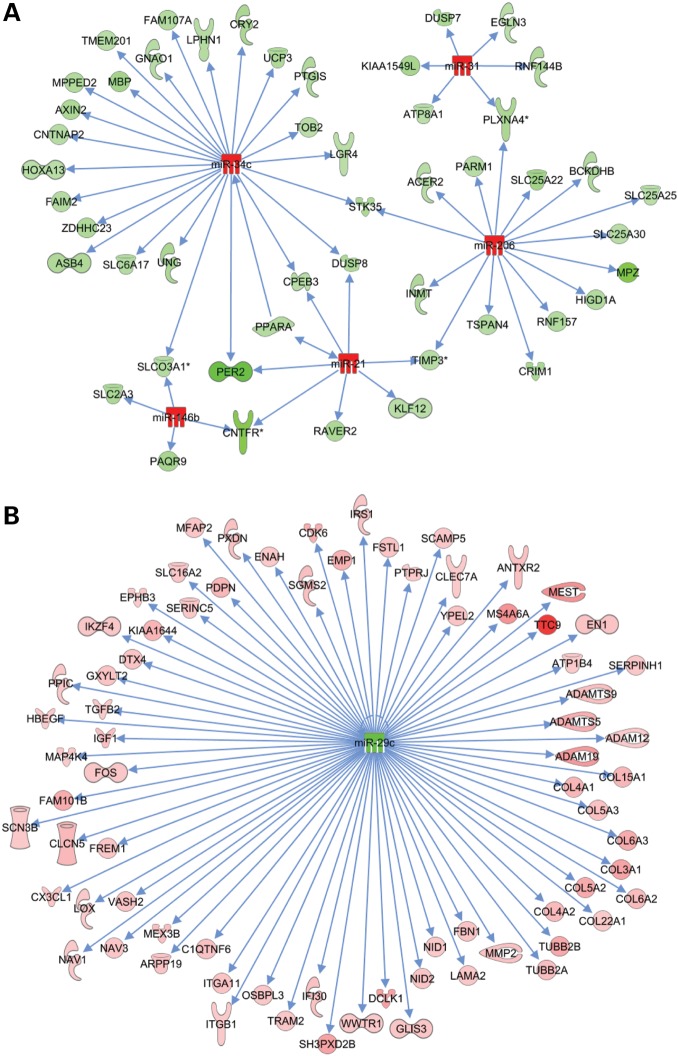
Identification of miRNA–mRNA target interactions. Network of miRNA–mRNA interactions identified by IPA for (**A**) dystromiRs up-regulated in *mdx* muscle (miR-21, miR-31, miR-34c, miR-146b and miR-206), and (**B**) the dystromiR miR-29c, which is down-regulated in *mdx* muscle. Red indicates increased expression in dystrophic muscle and green indicates reduced expression.

To identify possible functions for differentially expressed miRNAs in dystrophic muscle, we used the MicroRNA-Target Filter function in IPA to integrate the miRNomics and gene expression datasets. Analysis was limited to the six dystromiRs discussed earlier in order to focus on the interactions likely to be most relevant to dystrophic pathology (details of data filtering are given in Supplementary Materials and Methods). The resulting miRNA–mRNA interaction schemas with expression data overlaid are shown in Figure 6. When the same analysis was applied to the proteomics datasets, only four miRNA–target interactions were identified as a consequence of the narrower range of expression values for proteins relative to mRNAs, and due to incomplete proteome coverage. Two of these interactions, regulation of *Dmd* by miR-31 and *Dag1* (α/β-dystroglycan) by miR-21, can be excluded as in these cases, the cause of protein down-regulation is known to not be miRNA-mediated. The remaining interactions; regulation of *Tubb2a* by miR-29c and *Mpz* (myelin protein zero) by miR-206 were in agreement with the miRNA–mRNA target analysis. Taken together, these analyses have identified a set of likely miRNA–target interactions associated with the dystrophic phenotype.

## Discussion

Loss of functional dystrophin protein results in widespread proteomic, transcriptomic and miRNomic changes consistent with multiple secondary pathologies downstream of the primary genetic insult. Antisense oligonucleotide-mediated exon skipping restores dystrophin protein expression with concomitant shifts in the dystrophic proteome and transcriptome towards more wild-type-like expression profiles. In this study, we have utilized HiRIEF–LC-MS/MS proteomics to undertake the highest resolution analysis of differential protein expression in a DMD model reported to date (3272 proteins were quantified across two proteomics experiments). A major technical advantage of our study is the use of HiRIEF fractionation of peptides that simplifies the peptide mixture and attenuates the masking effect of peptides from highly-abundant proteins. Despite the depth achieved in our analysis, it is likely that we are still far from complete proteome coverage with many proteins remaining invisible to our analysis. Further methodological optimization will likely enable higher resolution analyses.

This study provides further evidence of the efficacy of exon skipping activity by P-PMO conjugates by demonstrating partial restoration of dystrophin protein and the dystrophic proteome/transcriptome consistent with the partial correction of DMD-associated secondary pathologies. In this study, we chose to investigate the effect of only a single dose of P-PMO as we have previously shown that this was sufficient to induce moderate dystrophin expression and restoration of circulating miRNA biomarkers to wild-type levels (13,36). We have subsequently shown that repeat administration of P-PMO conjugates induces a greater restoration in dystrophin expression (55,56) and so would also be expected to induce more complete correction of dystrophy-associated gene expression changes. Importantly, the level of dystrophin rescue possible in murine models is likely to be much higher than that achievable in patients with the current generation antisense oligonucleotide drugs. As a result, the more modest dystrophin correction observed after a single administration is likely to more accurately model the expected clinical situation.

A previous microarray study of antisense oligonucleotide-treated *mdx* muscle showed negligible restoration of dystrophic gene expression changes (57). In contrast, exon skipping mediated by the U7-snRNA system after viral delivery achieved higher levels of dystrophin rescue and a dose-dependent restoration of the dystrophic transcriptome. Notably, for methodological reasons this study defined restoration according to changes in a pre-defined set of genes known to be differentially expressed in young *mdx* muscle (57), whereas the present study treats the detectable proteome and transcriptome in an unbiased manner. mRNAs that were restored towards wild-type levels in our dataset consistent with the findings of 't Hoen *et al*. include: *Bgn*, *Lgmn*, *Tyrobp*, *Timp1*, *Cd52*, *Lgals3*, *Itgb2*, *Tuba1c* and *Cd68* (57). Conversely, there was little similarity between our gene expression data and that reported in a separate study which profiled gene expression changes in *mdx* muscle after prednisolone treatment (58). This is unsurprising given that these two therapeutic approaches (exon skipping and corticosteroid treatment) are assumed to have different mechanisms of action.

The investigation of differential mRNA and protein expression revealed that, in general, more genes were up-regulated than down-regulated in *mdx* TA (Figs. 1 and 4). Similarly, experimental groups were easily distinguished based on their gene expression patterns, which underlines the biomarker potential of specific gene expression signatures. Some of the most promising tissue biomarkers (based on differential expression and concordant protein/mRNA levels) are summarized in Supplementary Material, Table S2.

We observed that multiple DAPC proteins were down-regulated in *mdx* tissue, whereas expression of their corresponding mRNAs was unaffected, consistent with the notion that mislocalization of these proteins leads to a reduction in their stability. Although these proteins have been previously identified as down-regulated in dystrophic muscle (59–62), no single unbiased proteomics study has been able to detect and quantify these proteins until now.

Consistent with previous studies, we have observed up-regulation of dystromiRs (e.g. miR-31, miR-34c and miR-206) in *mdx* TA (36,63). Interestingly, P-PMO treatment did not restore the pattern of dystromiR expression in dystrophic muscle towards wild-type levels suggesting that the contribution of miRNA-regulation to dystrophy-associated changes in protein output is limited, or alternatively, that failure to restore wild-type patterns of miRNA expression serves to limit proteomic restoration.

Multiple studies have reported poor correlations between proteomic and transcriptomic data (23–26). There are a number of possible biological explanations for these observations including (i) differential rates of mRNA/protein degradation, (ii) differential rates of transcription/translation, (iii) *trans*-acting regulatory factors (e.g. miRNAs and RNA binding proteins) and (iv) transcriptional ‘bursting’ which serves to ‘top up’ a relatively stable pool of cellular proteins (64). Conversely, technical issues may account for the differences in protein and mRNA expression analyses. Many integrated omics analyses have been performed in cell cultures (often from experiments performed at different times or on publicly available gene expression datasets generated by separate groups) (26). In these cases, slight differences between implementation of the experiment will likely reduce the correlation between transcriptome and proteome. In the present *in vivo* study, all analyses were performed on serial muscle cryosections taken from the same set of animals, thereby eliminating this source of ambiguity. Here, we observed a statistically significant but weak positive correlation between mRNA and protein levels consistent with previous reports (23–26). The notion of a one-to-one relationship between transcription and translation is clearly too simplistic, and exemplifies the value of integrated proteomics/transcriptomics studies.

Top candidate genes for further study were identified which exhibited concordant differential expression at both the protein and mRNA level (Fig. 4F, Supplementary Material, Table S2). Relatively little, or in some cases nothing, is currently known about the role of these genes in the generation of the DMD phenotype (examples discussed later). Several of these genes have been previously identified as differentially expressed in *mdx* muscle by DNA microarray (65) but until now, no protein level information has been available.

Notably, multiple myosin proteins were differentially expressed in *mdx* muscle which likely reflects a shift in fibre composition associated with increased muscle regeneration in dystrophic tissue, i.e. down-regulation of Myh2 and Myl3 (associated with slow-twitch fibres) in *mdx* muscle and up-regulation of Myl4 (expressed in embryonic muscle) and Myl6b (slow-twitch Myosin light chain). Another myofibrillar protein, myozenin 2 (Myoz2), was also down-regulated in *mdx* TA. This sarcomeric protein localizes to the Z-line and is believed to be involved in calcineurin signalling (66). The calcium binding protein S100a4 was the most up-regulated candidate gene at both the protein and transcript level. Furthermore, IPA revealed that all of the known down-stream targets of S100a4 were also up-regulated, consistent with increased S100a4 signalling activity (Supplementary Material, Fig. S15). S100a4 has been implicated in a wide variety of processes including metastasis, cell migration/invasion, angiogenesis, epithelial-mesenchymal transition, fibrosis, inflammation and myocardial injury (67–70) although it has not previously been implicated in dystrophic pathology. A similarly up-regulated gene, *Postn* (periostin) is known to be positively regulated by Tgfb1 and is expressed in fibroblasts and extracellular matrix in muscle (71). *Postn* is dynamically regulated during cardiotoxin-induced muscle injury and during embryogenesis suggesting a possible role in dystrophic muscle regeneration (71).

Several of the top candidate genes encode metabolic proteins involved in lipolysis (*Ces1d*), fatty acid metabolism (*Bdh1*), acetyl CoA synthesis (*Acss1*), cytochome P450 (*Cyp4f39*) and lactate dehydrogenase (*Ldhb*). Furthermore, signalling by the mitochondrial transcription factor Tfam was identified as being down-regulated in both proteomic and transcriptomic datasets. These specific findings fit well with the identification of Mitochondrial Dysfunction as a highly significantly perturbed canonical pathway in dystrophic muscle (Supplementary Material, Fig. S12A) (72–74).

Consistent with the identification of Axonal Guidance Signalling as a differentially regulated canonical pathway (Supplementary Material, Fig. S12C), we also found that many genes (predominantly) expressed in neurons, or primarily associated with neuronal functions, were among those highly changed in dystrophic muscle. These include *Ncam1*, *Pak1*, *Prune2*, *Efna2* and the β-tubulins *Tubb2a* and *Tubb6* (75–78). The increase in expression of these neuron-associated genes might follow as a consequence of the cycles of degeneration and regeneration observed in dystrophic muscle. Myofibres lacking neuronal control undergo atrophy (79), and so newly formed fibres would necessarily require innervation by a lower motor neuron, either by fusing with existing innervated fibres or by generating new neuromuscular junctions (NMJs) (requiring neuronal migration and neurite outgrowth). These gene expression changes may reflect the physiological process of new fibre innervation, or may represent dystrophin loss-associated NMJ defects as reported previously (80,81). However, we cannot currently exclude the alternative possibility that some of these so-called ‘neuronal-specific’ genes may also be expressed and have additional functions in skeletal muscle cells.

The integrated analysis of multiple omics experiments described here provides the most complete picture of gene expression in dystrophic muscle to date and provides fresh insights into the molecular biology of dystrophinopathy and the response to exon skipping therapy.

## Materials and Methods

### Animals

Male C57/Bl10 or C57/Bl10ScSn-*Dmd^mdx^*/J (*mdx*) mice were sacrifice at 8 or 14 weeks of age as appropriate and exsanguinated via the jugular vein. Treated animals were injected with a single 12.5 mg/kg dose of Pip6e-PMO in sterile saline via the tail vein at 12 weeks of age. TA muscles were macrodissected and snap frozen in liquid nitrogen-cooled isopentane. Cryosections (8 µm) were prepared from the mid-belly of the muscle and placed in separate tubes for each analysis.

### iTRAQ LC-MS/MS proteomics

Samples were reduced using dithiothreitol and alkylated with iodoacetamide followed by overnight trypsinization (Promega, Stockholm, Sweden). Peptides were iTRAQ labelled, pooled and separated by immobilized pH gradient—isoelectric focusing (IPG-IEF) on narrow range pH 3.7–4.9 and 4.00–4.25 strips, as described previously (21). Extracted fractions from the IPG-IEF were separated using an Agilent 1200 nano-LC system coupled to Thermo Scientific LTQ Orbitrap Velos. Proteome discoverer 1.3 with Sequest-percolator was used to search the UniProt mouse (120 524) database for protein identification, limited to a false discovery rate of <1%.

### Microarray analysis

Total RNA (500 ng or 100 ng) was hybridized to GeneChip miRNA 3.0 arrays or Gene ST 1.1 Array plates, respectively (both Affymetrix, Santa Clara, CA, USA). All microarray data are accessible through GEO Series accession number GSE64420.

### Bioinformatics

Proteomics and transcriptomics data were analysed by *t*-test (permutation setting, FDR correction), one-way ANOVA (Bonferroni correction), hierarchical clustering, PCA and volcano plot using TMeV (Institute for Genomic Research, Rockville, MD, USA) (82). Additional analyses were performed in GraphPad Prism 5 (GraphPad Software Inc., La Jolla, CA, USA). Pathway analysis was performed using IPA and gene list enrichment with ToppGene (83).

## Supplementary Material

Supplementary Material is available at *HMG* online.

## Funding

T.C.R. is supported by Medical Research Council UK Centenary Early Career Award. Work in the laboratory of M.J.G. was supported by the Medical Research Council (MRC programme number U105178803). Work in the laboratory of C.I.E.S. is supported by the Swedish Research Council. Funding to pay the Open Access publication charges for this article was provided by the Medical Research Council UK.

## Supplementary Material

Supplementary Data
