# Supplementary material for: Multi-level omics analysis in a murine model of dystrophin loss and therapeutic restoration
Source: Hum Mol Genet. 2015 Sep 18;24(23):6756–68. doi: 10.1093/hmg/ddv381 (PMC4634378; doi:10.1093/hmg/ddv381)
Supplement: Supplementary Data [file supp_24_23_6756__index.html]

Multi-level omics analysis in a murine model of dystrophin loss and therapeutic restoration — Multi-level omics analysis in a murine model of dystrophin loss and therapeutic restoration — Multi-level omics analysis in a murine model of dystrophin loss and therapeutic restoration — Supplementary Data 

# Multi-level omics analysis in a murine model of dystrophin loss and therapeutic restoration

## Supplementary Data

Supplementary Data

- Supplementary Data - Docx file
