# Supplementary material for: Multi-level omics analysis in a murine model of dystrophin loss and therapeutic restoration
Source: Hum Mol Genet. 2015 Sep 18;24(23):6756–68. doi: 10.1093/hmg/ddv381 (PMC4634378; doi:10.1093/hmg/ddv381)
Supplement: Supplementary Data [file supp_ddv381_ddv381supp.docx]

**Supplementary Figure Legends**

**
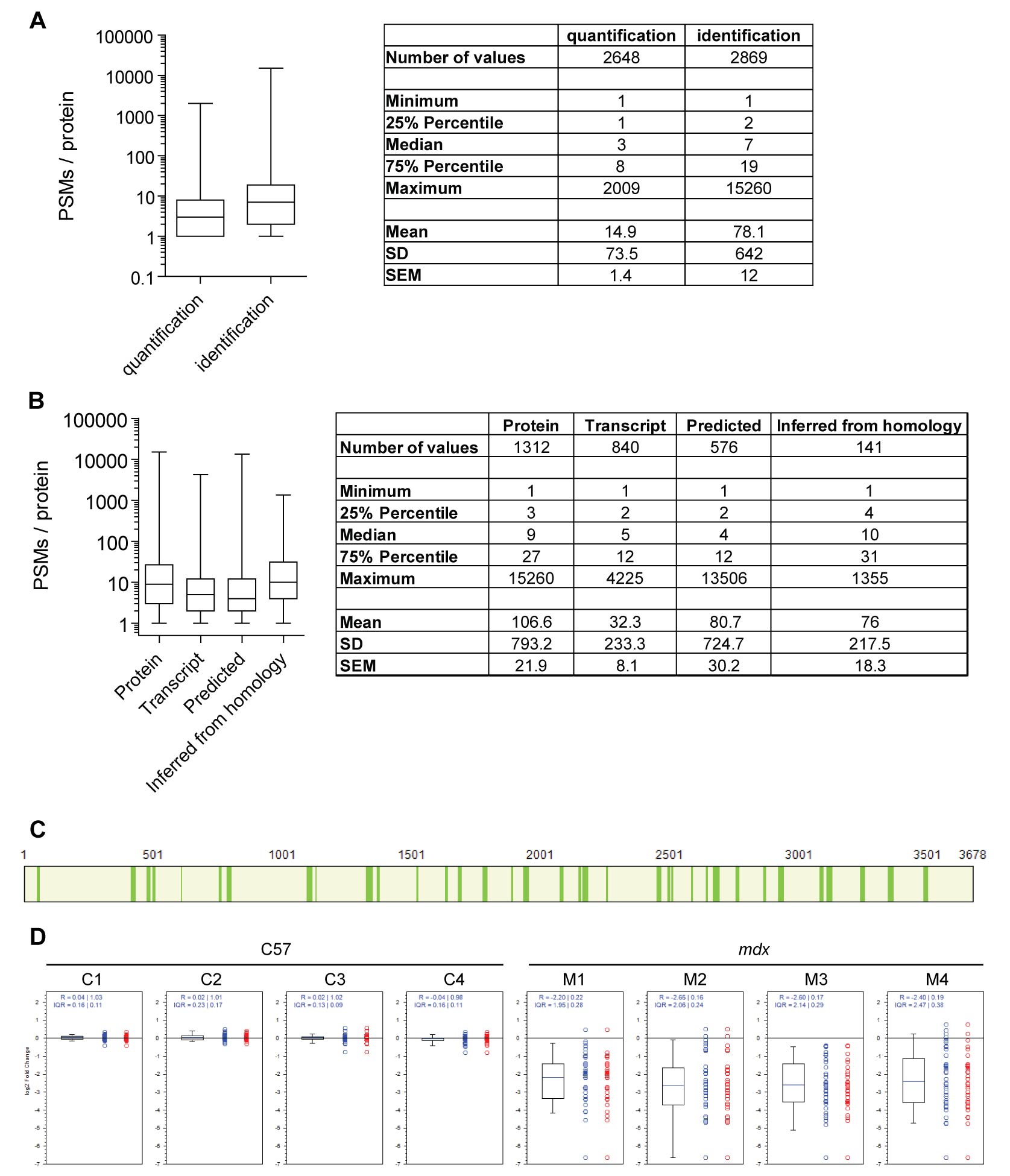
**

**Figure S1**

**Peptide statistics for 8 week old C57 vs *mdx* experiment.**

(a) Distribution of peptide spectrum matches (PSMs) per protein, used for protein quantification and identification. (b) PSM distribution for proteins sorted by levels of evidence. (c) Peptide sequence coverage for dystrophin. Green lines indicate identified peptides and their locations. Numbers indicate amino acid positions. (d) Distribution of dystrophin peptide ratios used for quantification. Box plot and blue circles show peptide ratio distribution of peptides used for quantification. Peptide ratios in red are not used for quantification (e.g. the peptide sequence was not unique for dystrophin).

**
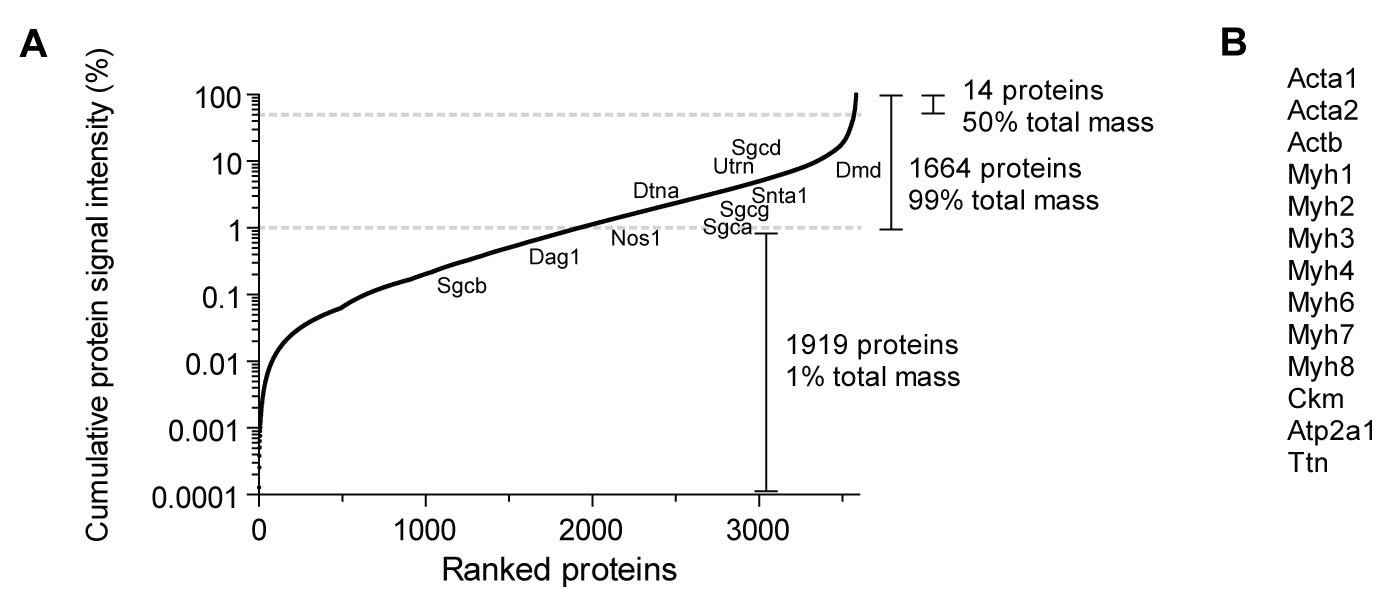
**

**Figure S2**

**Estimation of protein proportion and dynamic range in muscle tissue.**

PSM data for both the 8 week old and 14 week old proteomics experiments was pooled and proteins ranked by number PSMs/protein. The percentage of the total protein signal was estimated for each protein by dividing the number of PSMs/protein by the total number of PSMs. The resulting data are shown in the cumulative frequency plot (a). 14 proteins make up 50% of the total mass. The top 1,664 proteins comprised 99% of the total mass (including the majority of the DAPC components). The 1,919 least abundant proteins comprised only 1% of the total mass. (b) The identities of the most abundant proteins are listed. (Note: This list contains two isoforms of Ttn although the protein symbol is listed only once).

**
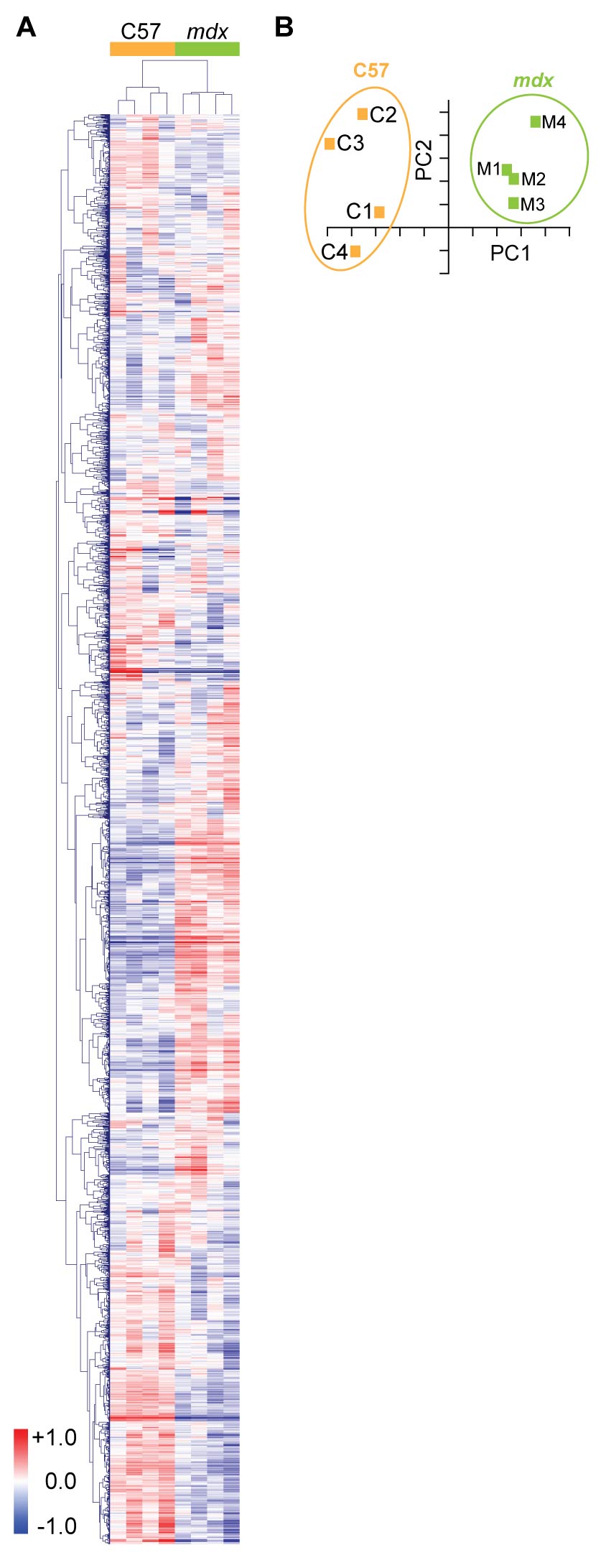
**

**Figure S3**

**Unsupervised analyses of protein expression ratios.**

Protein expression ratios from 8 week old C57 vs *mdx* tibialis anterior samples were analysed by (a) unsupervised hierarchical clustering and (b) principal component analysis (the first two components represent 63% of the data). Red indicates up-regulated proteins and blue indicates down-regulated proteins. The scale bar represents the row Z-score.

**
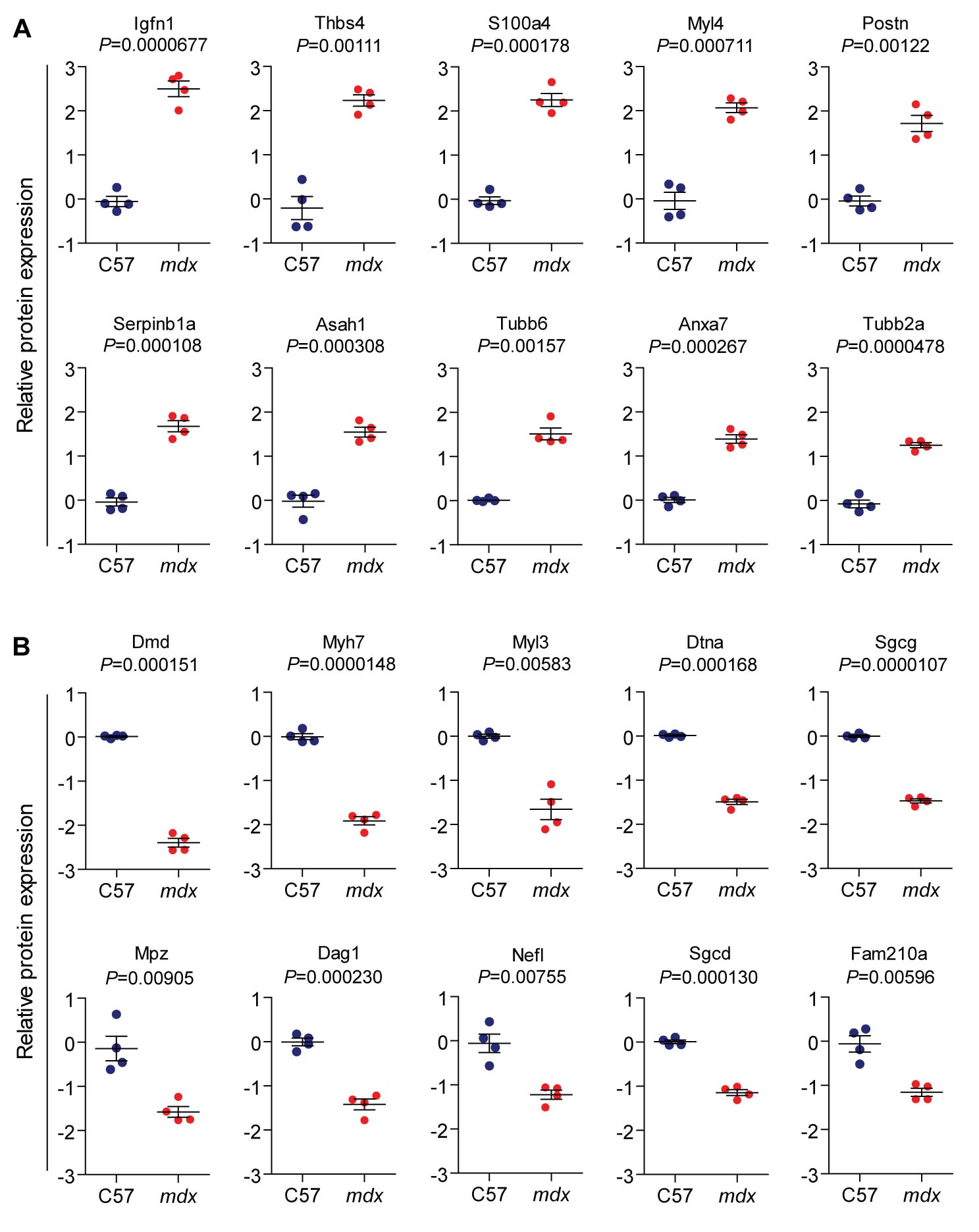
**

**Figure S4**

**Most differentially expressed proteins in 8 week old *mdx* tibialis anterior.**

The 10 most (a) up-regulated and (b) down-regulated proteins in 8 week old C57 vs *mdx* tibialis anterior muscles. Individual log_2_ expression ratios for each replicate are shown. The mean and SEM values are indicated. *P* values are shown for each protein.

**
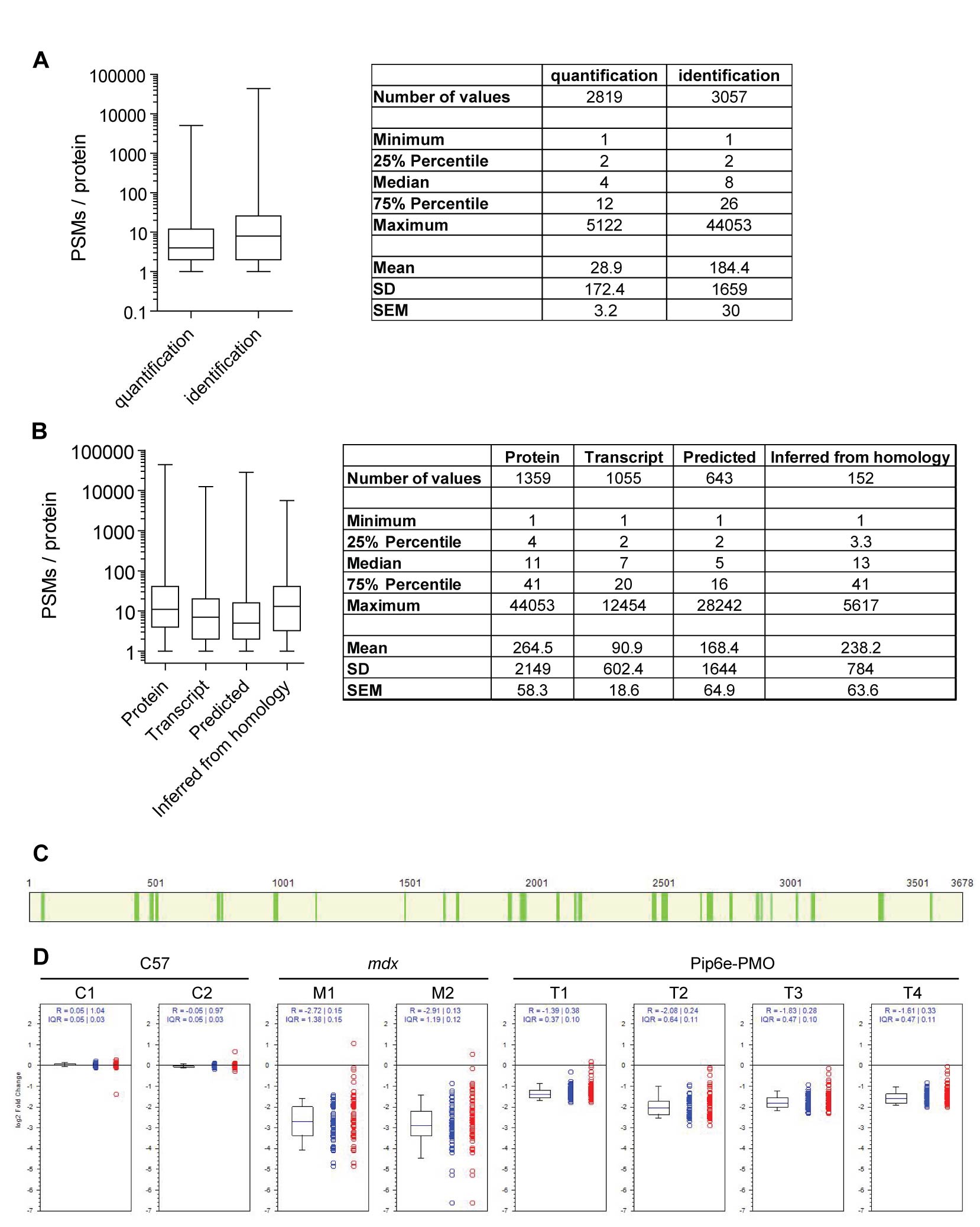
**

**Figure S5**

**Peptide statistics for 14 week old C57 vs *mdx* vs Pip6e-PMO-treated *mdx* experiment.**

(a) Distribution of peptide spectrum matches (PSMs) per protein for protein quantification and identification. (b) PSMs per protein for proteins sorted by levels of evidence. (c) Peptide sequence coverage for dystrophin protein. Green lines identified peptide locations. Numbers indicate amino acid positions. (d) Distribution of dystrophin peptides ratios used for quantification. Box plot and blue circles show peptide ratio distribution of peptides used for quantification. Red circles represent peptide ratios that were not used for quantification (e.g. the peptide sequence was not unique for dystrophin).

**
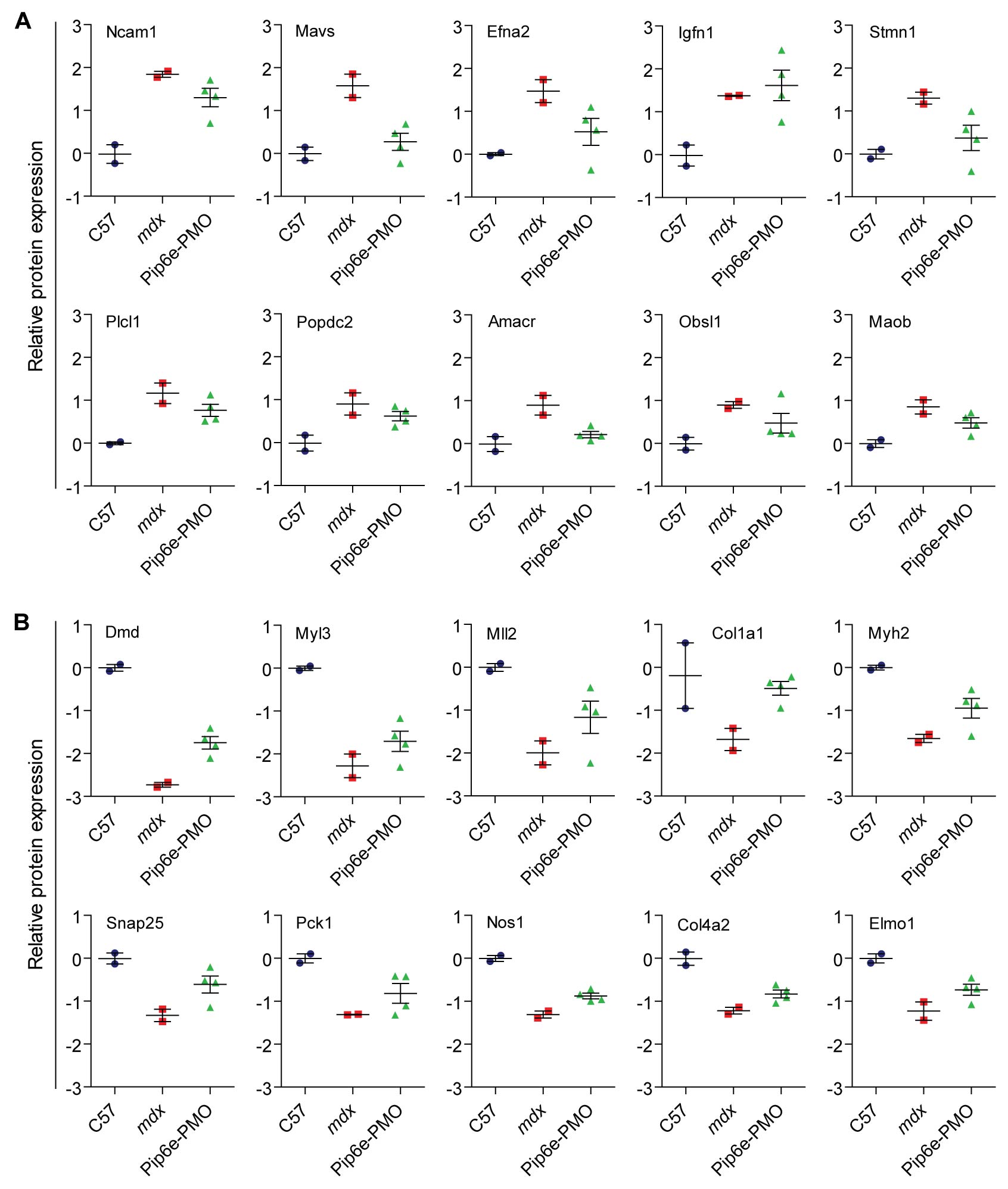
**

**Figure S6**

**Most differentially expressed proteins in 14 week old *mdx* tibialis anterior muscles.**

The 10 most (a) up-regulated and (b) down-regulated proteins in 14 week old C57, *mdx* and Pip6e-PMO-treated *mdx* tibialis anterior muscles. Individual log_2_ expression ratios for each replicate are shown. The mean and SEM values are indicated.

**
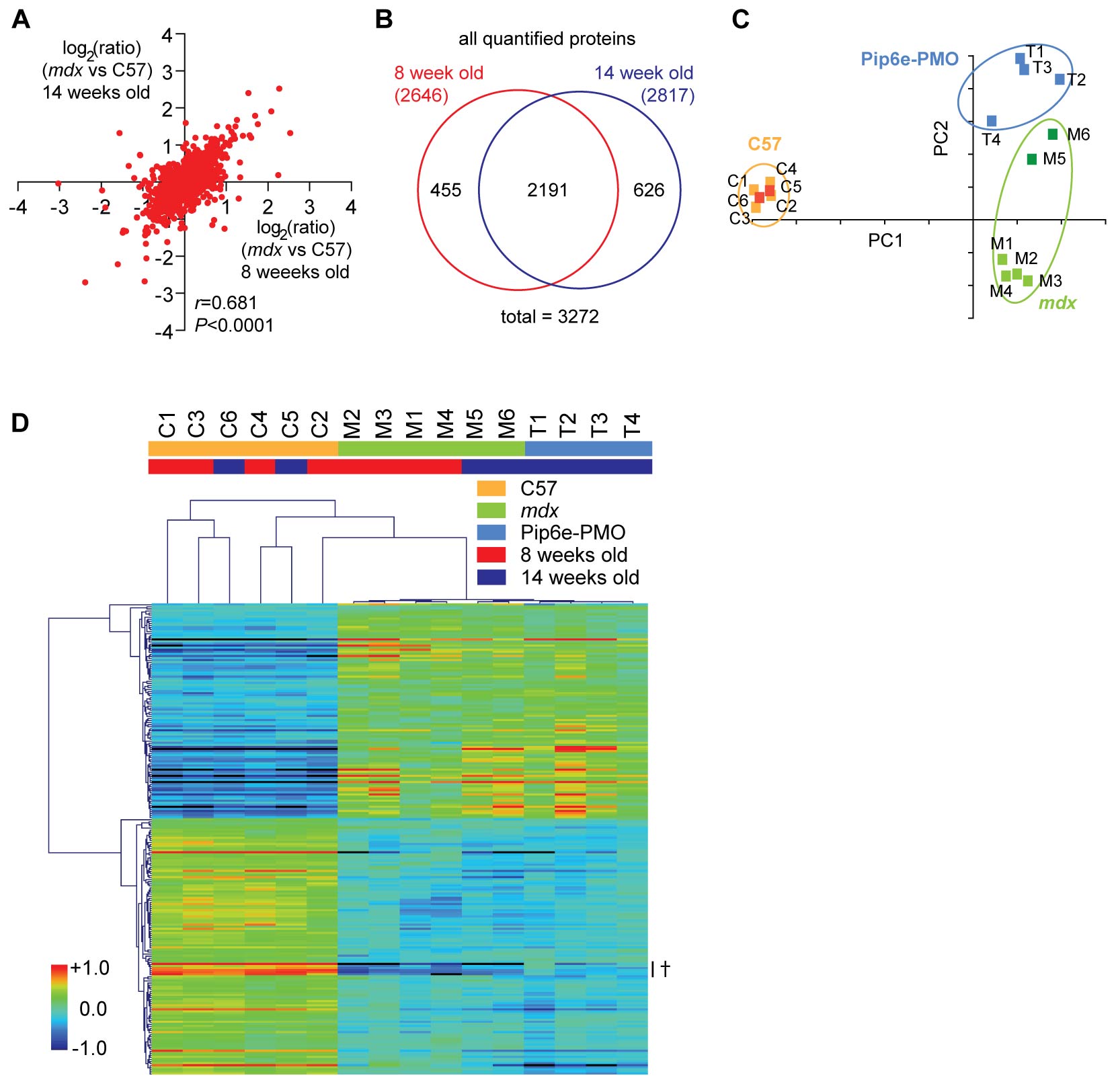
**

**Figure S7**

**Comparison of data from two iTRAQ LC-MS/MS proteomics experiments.**

(a) Similarities between proteomics datasets were assessed by plotting log_2_ expression ratios against one another. A highly significant positive correlation between datasets was observed. (b) Venn diagram showing overlap between all quantified proteins between both LC-MS/MS proteomics experiments (8 week old and 14 week old mice). Expression ratios from both experiments were combined and analysed by (c) principal component analysis (the first two components representing 92.4% of the data are shown), and (d) unsupervised hierarchical clustering. Red and blue indicate up- and down-regulated protein expression respectively. Green and light blue indicate intermediate protein expression levels. The scale bar represents the row Z-score. † indicates a cluster of genes-of-interest which showed restoration following treatment with Pip6e-PMO. Both analyses correctly clustered samples according to experimental group.

**
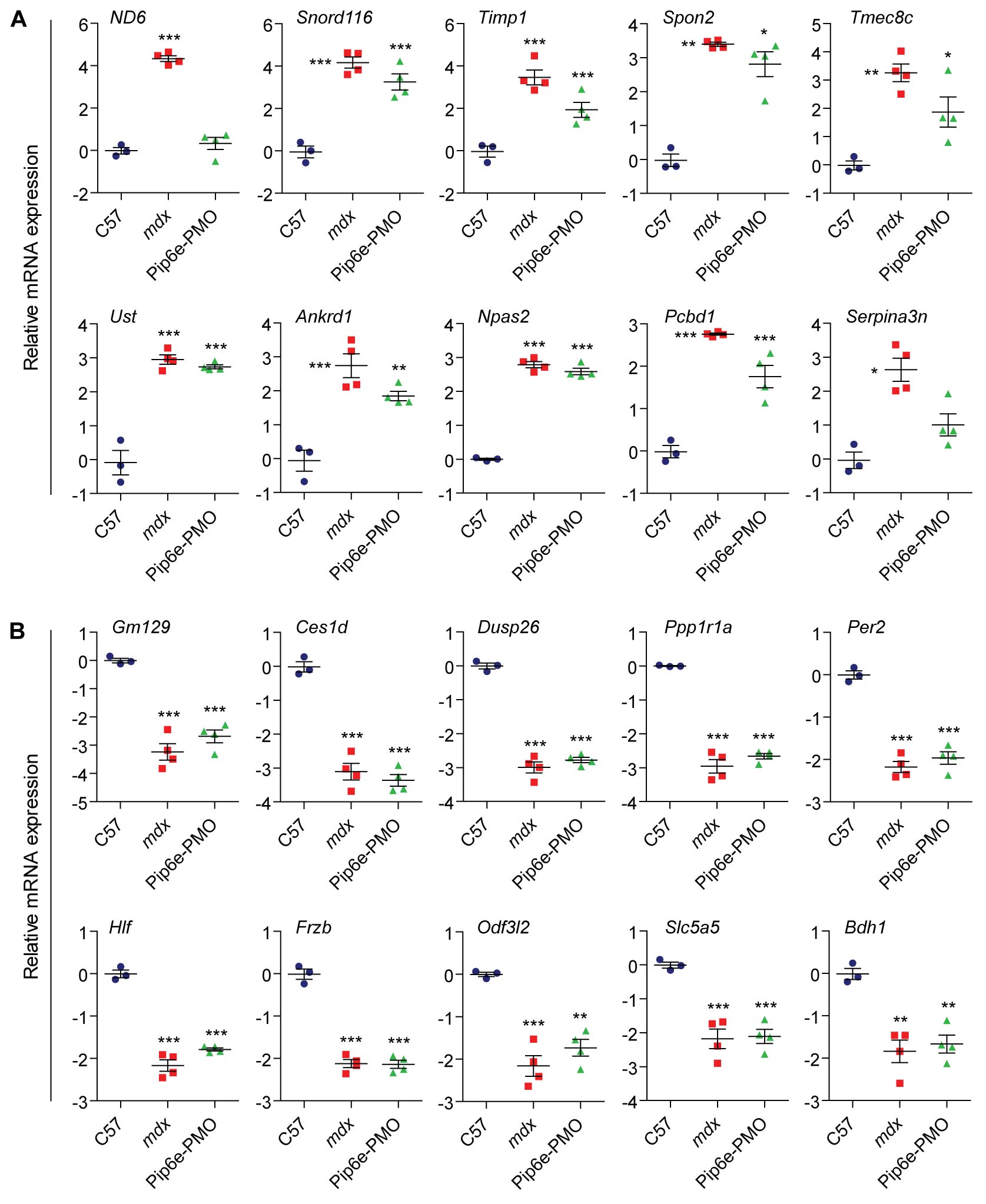
**

**Figure S8**

**Most differentially expressed mRNAs in 14 week old *mdx* tibialis anterior.**

The 10 most (a) up-regulated and (b) down-regulated mRNAs in 14 week old C57, *mdx* and Pip6e-PMO-treated *mdx* tibialis anterior muscles. Individual log_2_ expression ratios for each replicate are shown. The mean and SEM values are indicated. Statistical comparisons are relative to C57 control group. **P*<0.05, ***P*<0.01, *** *P*<0.001, One way ANOVA with Bonferroni *post hoc* test.

**
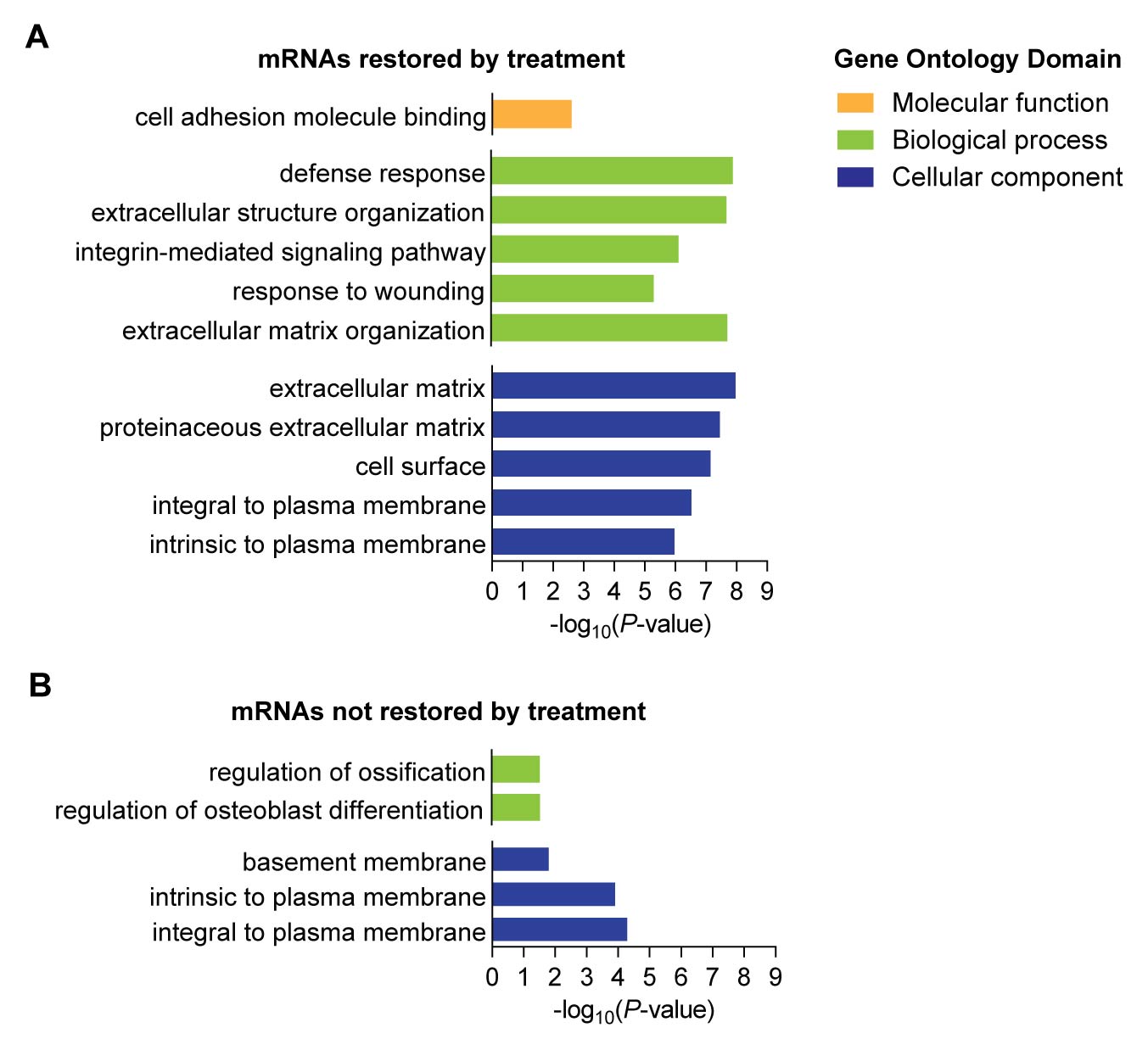
**

**Figure S9**

**Gene ontology analysis of mRNA expression in response to exon skipping therapy.**

(a) Gene ontology terms enriched in the set of mRNAs that were (a) restored, or (b) not restored towards wild-type levels in *mdx* mice following treatment with Pip6e-PMO.

**
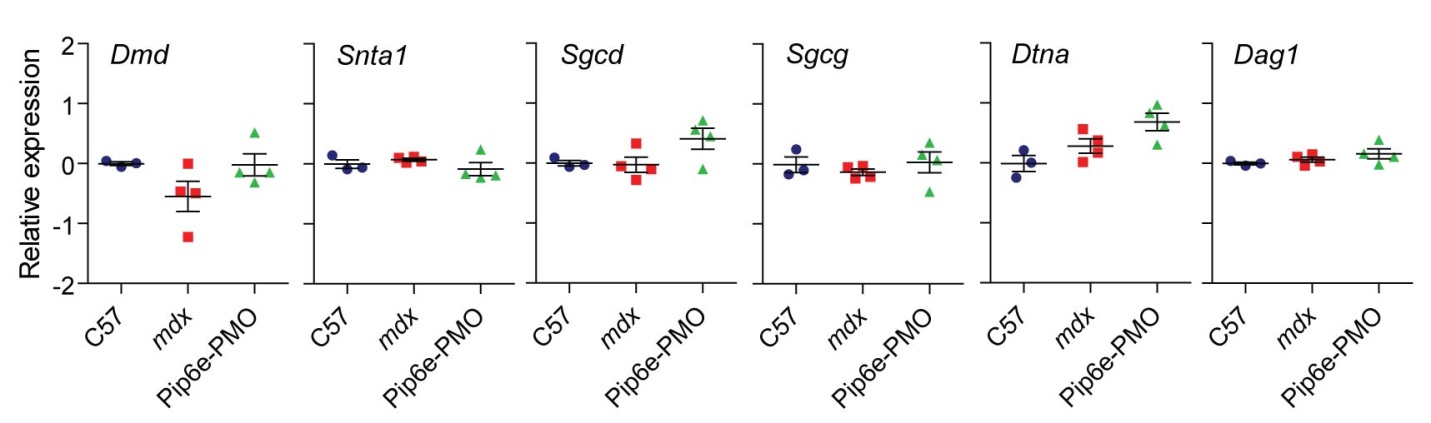
**

**Figure S10**

**mRNA expression for members of the DAPC.**

Individual log_2_ expression ratios for each replicate are shown. The mean and SEM values are indicated.

**
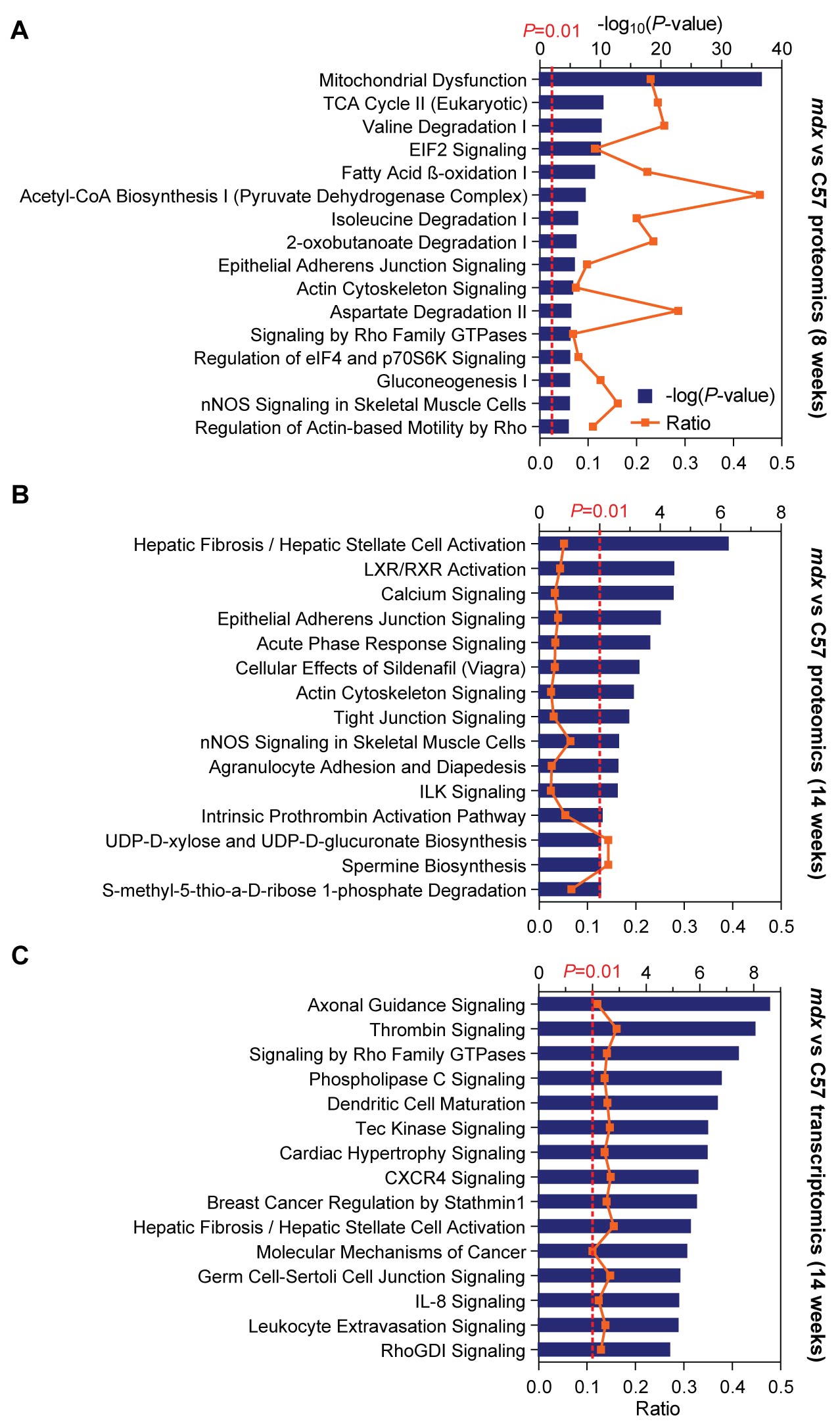
**

**Figure S11**

**Differentially regulated canonical pathways in dystrophic muscle.**

Canonical pathways that were differentially regulated in *mdx* muscle were identified by Ingenuity pathway analysis for (a) the 8 week old proteomics, (b) the 14 week proteomics, and (c) the 14 week transcriptomics datasets. The top scoring pathways were ranked by descending -log_10_*P* value. The ratio of number of genes affected to the total number of genes in a given gene set is indicated by orange lines. The dotted red line indicates the *P*<0.01 significance threshold.

(Fewer pathways were identified, and with lower significance, for the 14 week old proteomics experiment on account of replicate variability based-filtering leading to a reduced number of proteins analysed).

**
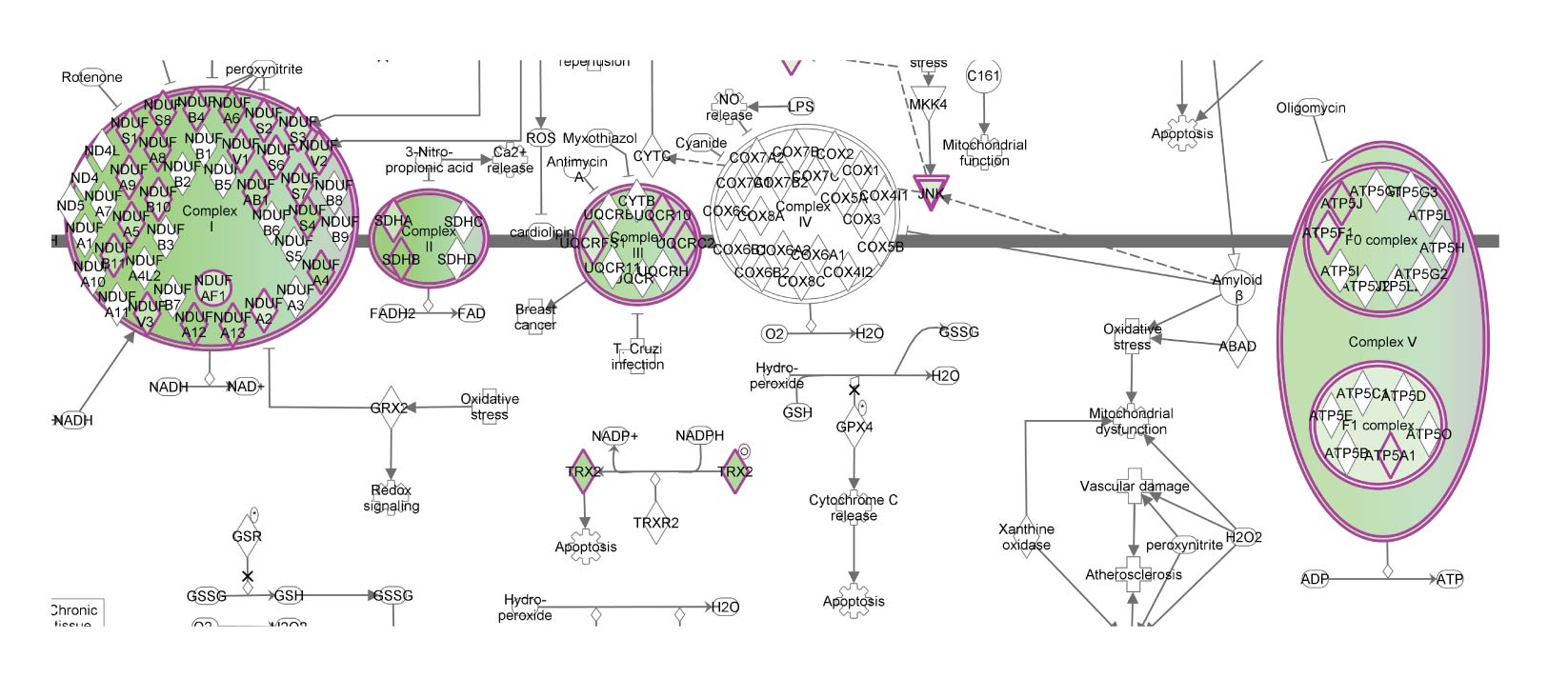
**

**Figure S12**

**Disruption of electron transport complexes in dystrophic muscle.**

Mitochondrial dysfunction was identified as a perturbed canonical pathway in *mdx* muscle. A section of the pathway is shown which includes the five electron transport complexes. Green indicates down-regulated protein expression

**
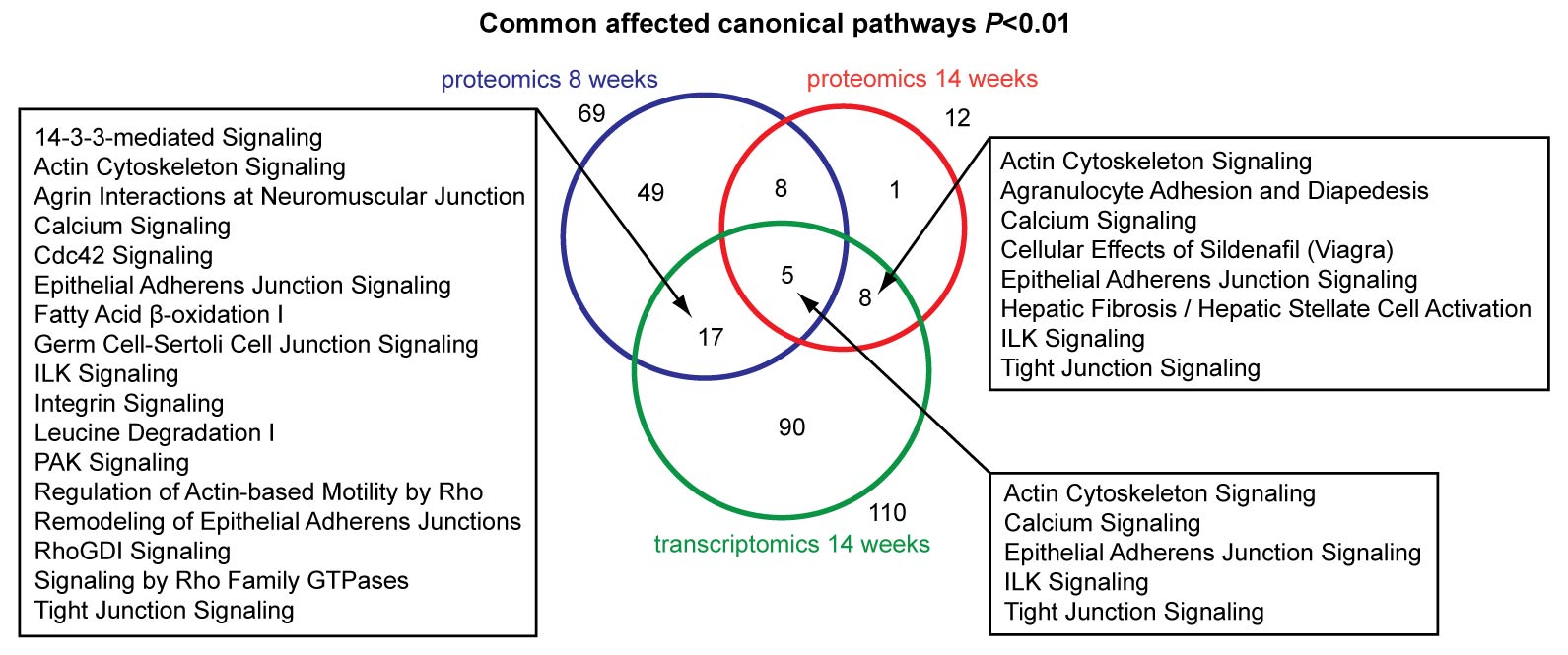
**

**Figure S13**

**Commonly affected canonical pathways in dystrophic muscle.**

Venn diagram indicating differentially regulated (*P*<0.01) canonical pathways between proteomics and transcriptomics datasets. 5 differentially regulated canonical pathways were common between all datasets: Actin Cytoskeleton Signalling, Calcium Signalling, Epithelial Adherens Junction Signalling, ILK Signalling, and Tight Junction Signalling.

**
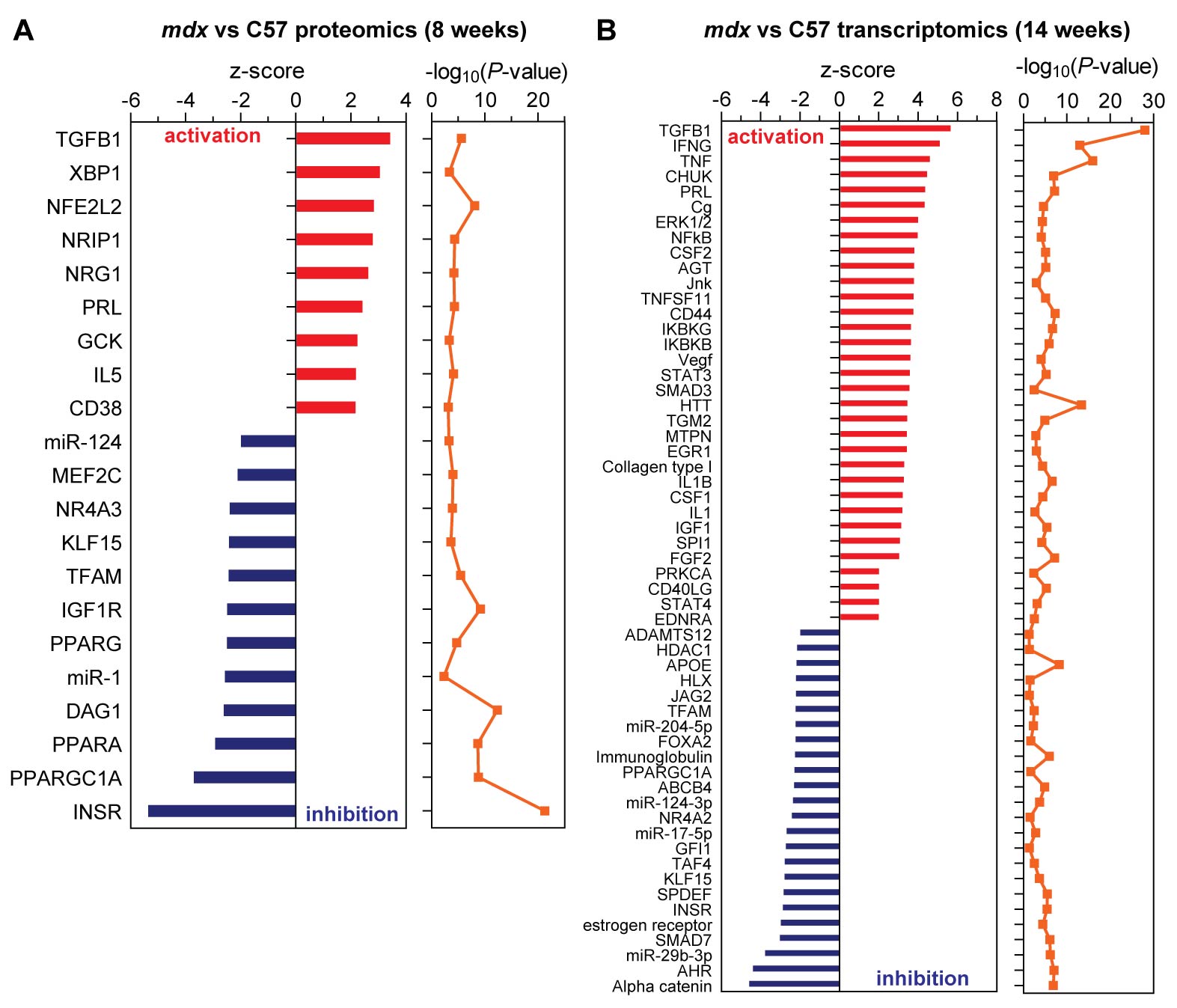
**

**Figure S14**

**Upstream regulator analysis in dystrophic muscle.**

Ingenuity pathway analysis was used to identify predicted upstream regulators that were differentially affected in *mdx* muscle based on the expression ratios of their known downstream targets for (a) the 8 week old proteomics and (b) 14 week transcriptomics datasets. Upstream regulators are ranked by z-score (positive values indicate activation and negative values indicate inhibition of the predicted regulator). Statistical significance is indicated by orange lines. Six regulators were predicted to be differentially affected in both datasets: INSR, KLF15, PPARGC1A, PRL, TFAM and TGFB1. (Note: IPA produces output with human gene identifiers).

**
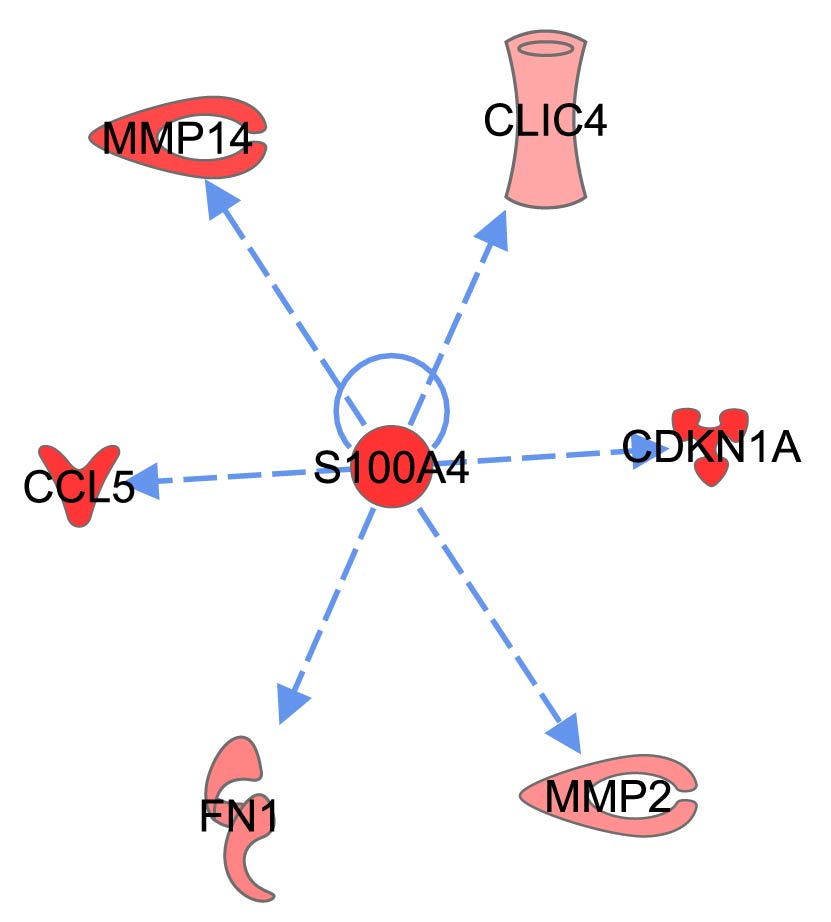
**

**Figure S15**

**Downstream targets of S100a4 are concordantly activated.**

S100a4 is up-regulated in dystrophic muscle at both the protein and mRNA level. IPA was used to identify downstream targets of S100a4 and mRNA expression ratios overlaid on the pathway. 6 target mRNAs that are known to be up-regulated by S100a4 were also found to be up-regulated in *mdx* muscle, consistent with S100a4 activation. Red indicates increased expression in dystrophic muscle.

**Supplementary Tables**

| **Methodology** | **Experimental Design** | **Number of changed proteins** | **Comments** | **Citation** |
| --- | --- | --- | --- | --- |
| 2DE | C57 vs *mdx*  3 months  Hindlimb | 60 | Reduced adenylate kinase 1 expression | (1) |
| 2DE | C57 vs *mdx*  9 weeks | 8 of 11 | Reduction Ca2+ buffering proteins (i.e. Calsequestrin, Sarcalumenin) | (2) |
| 2DE | C57 vs *mdx*  1,3 6 months  Hindlimb | 24 | Myosin light chain 2 up-regulated at all ages | (3) |
| 2DE | C57 vs *mdx*  diaphragm | 20 | Down-regulated regucalcin | (4) |
| 2D-DIGE | Normal vs *mdx*  Diaphragm | 35 | 35 changed  cvHSP up-regulated | (5) |
| 2D-DIGE | C57 vs *mdx*  1, 3, 5, 7, 9 months  Heart | 14 | Combined metabolomics and proteomics | (6) |
| 2D-DIGE | C57 vs *mdx* vs PMO | 20 | 20 changed  AK1 restoration | (7) |
| 2D-DIGE | C57 vs *mdx*  9 weeks  Extraocular muscles | 7 | 7 changed | (8) |
| 2D-DIGE | C57 vs *mdx*  9 months  Heart | 29 |  | (9) |
| 2D-DIGE | C57 vs *mdx*  6 weeks  Gastrocnemius | 61 | Acute phase | (10) |
| iCAT | GRMD vs healthy dogs  4 months  Vastus lateralis | 84 | PGC1α down  Cytosolic and phospho-enriched proteins | (11) |
| 2D-DIGE | C57 vs *mdx*  8 weeks, 12, 22 months  Diaphragm | 11 | Aging associated changes in extracellular matrix proteins | (12) |
| 2D-DIGE | C57 vs *mdx*  8 weeks, 12, 22 months | 8 | CA3 isoform of carbonic anhydrase increase and Hsp72 decreased | (13) |
| IP-LC-MS/MS | C57 vs *mdx* | NA | Immunoprecipitation of DAPC | (14) |
| SILAC mouse | C57Bl/6 vs *mdx*-52  3 weeks  Gastrocnemius | 73 | 789 quantified  ILK pathway and actin cytoskeleton perturbed | (15) |

**Table S1**

**Summary of Proteomics Studies in Dystrophic Muscle.**

2DE, Two-Dimensional gel Electrophoresis. 2D-DIGE, Two-Dimensional-Difference Gel Electrophoresis. iCAT, isotope-Coded Affinity Tag. SILAC mouse, Stable Isotope Labelling by Amino acids in Cell culture – adapted for *in vivo* mouse use. IP-LC-MS/MS, Immunoprecipitation followed by Liquid Chromatography-tandem Mass Spectrometry.

| ***mdx* vs C57**  **Proteomics**  **(8 weeks)** | ***mdx* vs C57**  **Proteomics**  **(14 weeks)** | ***mdx* vs C57**  **Transcriptomics**  **(14 weeks)** | ***mdx* vs C57**  **Concordant protein and mRNA** | ***mdx* vs C57**  **miRNomics**  **(14 week)** |
| --- | --- | --- | --- | --- |
| **Dmd** | **Dmd** | Gm129 | Cmbl | miR-551b* |
| Myh7 | Mll2 | **Ces1d** | **Ces1d** | miR-381* |
| **Myl3** | **Myl3** | Dusp26 | **Myl3** | miR-434-3p |
| Dtna | Col1a1 | Ppp1r1a | 5730469M10Rik | miR-101b |
| Sgcg | **Myh2** | Per2 | **Myh2** | miR-29c* |
| Mpz | Snap25 | Hlf | Ldhb | **miR-29c** |
| Dag1 | Pck1 | Frzb | Myoz2 | miR-539-5p |
| Nefl | Nos1 | Odf3l2 | Cyp4f39 | miR-337-5p |
| Sgcd | Col4a2 | Slc5a5 | Acss1 | miR-329 |
| Fam210a | Elmo1 | **Bdh1** | **Bdh1** | miR-181a-2* |
| **Igfn1** | **Igfn1** | ND6 | **Igfn1** | **miR-31** |
| Thbs4 | **Ncam1** | Snord116 | **Ncam1** | miR-31* |
| **S100a4** | Mavs | Timp1 | **S100a4** | **miR-34c** |
| **Myl4** | Efna2 | Spon2 | **Myl4** | **miR-206** |
| **Postn** | Stmn1 | Tmec8c | **Postn** | miR-501-3p |
| **Serpinb1a** | Plcl1 | Ust | **Serpinb1a** | miR-501-5p |
| Asah1 | Popdc2 | Ankrd1 | Ckap4 | miR-675-3p |
| **Tubb6** | Amacr | Npas2 | **Tubb6** | miR-34c* |
| Anxa7 | Obsl1 | Pcbd1 | Pak1 | miR-34b-3p |
| **Tubb2a** | Maob | Serpina3n | **Tubb2a** | **miR-146b** |
|  | | | Eef1a1 |  |
|  |  |  | Efna2 |  |
|  |  |  | Prune2 |  |
|  |  |  | Aspa |  |
|  |  |  | Myl6b |  |

**Table S2**

**Summary of most differentially expressed genes in *mdx* muscle.**

The top ten up- and down-regulated genes are shown for protein, mRNA and miRNA datasets. For the concordant protein and mRNA analysis all statistically significant genes (*P*<0.01) are shown that were differentially expressed by at least 2 fold as determined by one or both methods. Red indicates up-regulation and blue indicates down-regulation in *mdx* muscle. Proteins/mRNAs that are common between analyses are in bold and underlined. For miRNAs, dystromiRs that were identified as of key importance are highlighted in bold and underlined.

**Supplementary Materials and Methods**

**Animal Procedures**

All animal experiments were carried out in accordance to procedures authorised by the UK Home Office in accordance with UK law (i.e. Animals (Scientific Procedures) Act 1986). Pip6e peptide: Ac-RXRRBRRXYRFLIRXRBRXRB-OH (where X is aminohexanoyl and B is β-alanine) and PMO: 5′-GGCCAAACCTCGGCTTACCTGAAAT (Gene Tools LLC, Philomath, OR) were conjugated as described previously (16).

**Sample Preparation for Mass Spectrometry**

Serial sections of the tibialis anterior muscle were lysed in 4% SDS, 25 mM HEPES, 1 mM DTT, pH 7.6 and sonicated. Samples were mixed with 1 mM DTT, 8 M urea, 25 mM HEPES, pH 7.6 in a centrifugation filtering unit, 10 kDa cutoff (Nanosep Centrifugal Devices with Omega Membrane), and centrifuged for 15 minutes at 14,000 *g*, followed by another addition of the 8 M urea buffer and centrifugation. Proteins were alkylated by use of 55 mM iodoacetamide, in 8 M urea, 25 mM HEPES, pH 7.6 for 10 min, centrifuged, followed by 2 more additions and centrifugations with 8 M urea, 25 mM HEPES pH 7.6. Trypsin (Promega). 1:50, trypsin:protein, was added to the samples in 0.25 M urea, 25 mM HEPES and digested overnight at 37 °C. The filter units were centrifuged for 15 minutes at 14,000 *g*, followed by another centrifugation with Milli-Q water. Peptide flow-throughs were collected, iTRAQ labeled according to manufacturer’s instructions, pooled, and cleaned using a Strata-X-C-cartridge (Phenomenex).

**IPG-IEF of Peptides**

iTRAQ labelled peptides were separated by immobilized pH gradient - isoelectric focusing (IPG-IEF) on narrow range pH 3.7-4.9 and 4.00-4.25 strips as described by previously (17). Peptides were extracted from the IPG-IEF strips by a prototype liquid handling robot, kindly supplied by GE Healthcare Bio-Sciences AB. A plastic device with 72 wells was put onto each strip and 50 µl of Milli-Q water was added to each well. After 30 minutes incubation, the liquid was transferred to a 96 well plate and the extraction was repeated 2 more times. The extracted peptides were dried by speedvac and dissolved in 3% acetonitrile (ACN), 0.1% formic acid before analysis on MS.

**LC- LTQ-Orbitrap Analysis**

Before analysis on the LTQ Orbitrap Velos (Thermo Fischer Scientific, San Jose, CA, USA), peptides were separated using an Agilent 1200 nano-LC system. Samples were trapped on a Zorbax 300SB-C18, and separated on a NTCC-360/100-5-153 (Nikkyo Technos., Ltd) column using a gradient of A (3% acetonitrile (ACN), 0.1% formic acid (FA)) and B (95% ACN, 0.1% FA), ranging from 3% to 40% B in 50 minutes with a flow of 0.4 µl/min. The LTQ Orbitrap Velos was operated in a data dependent manner, selecting 5 precursors for sequential fragmentation by Collison-Induced Dissociation (CID) and Higher-energy Collisional Dissociation (HCD), and analyzed by the linear iontrap and orbitrap, respectively. The survey scan was performed in the Orbitrap at 30,000 resolution (profile mode) from 300-2000 *m/z,* using lock mass at *m/z* 445.120025, with a max injection time of 500 ms and Automatic Gain Control (AGC) set to 1 x 10^6^ ions. For generation of HCD fragmentation spectra, a max ion injection time of 500 ms and AGC of 5 x 10^4^ were used before fragmentation with 37.5% normalized collision energy. For Fourier Transform Mass Spectrometry (FTMS) MS2 spectra, normal mass range was used, centroiding the data at 7500 resolution. Peptides for CID were accumulated for a max ion injection time of 200 ms and AGC of 3x10^4^, fragmented with 35% collision energy, wideband activation on, activation q 0.25, activation time 10 ms before analysis at normal scan rate and mass range in the linear iontrap. Precursors were isolated with a width of 2 *m/z* and put on the exclusion list for 90 s. Single and unassigned charge states were rejected from precursor selection.

**Peptide and Protein Identification**

All Orbitrap data was searched by sequest-perculator under the software platform Proteome Discoverer 1.3 (Thermo) against UniProt mouse database (120524) and filtered to a 1% FDR. A precursor mass tolerance of 10 ppm, and product mass tolerances of 0.02 Da for HCD-FTMS and 0.4 Da for CID-ITMS were used. Further settings used were: trypsin with 1 missed cleavage; iodoacetamide on cysteine, iTRAQ on lysine and N-terminus as fixed modifications, and oxidation of methionine as variable modification. Quantization of iTRAQ8plex reporter ions was done by Proteome Discoverer on HCD-FTMS tandem mass spectra using an integration window tolerance of 20 ppm. Only unique peptides in the data set were used for quantification.

**Proteomics Analysis**

For the 8 week and combined analyses, protein expression ratios were filtered by *P* value and expression ratios were given relative to the C57 control. For the 14 week old proteomics dataset *n* numbers in *mdx* and C57 groups were too small to permit statistics based filtering. Instead, protein expression ratios were filtered based on replicate consistency for the denominator (*mdx*). First, potential outliers were removed by dividing the 2 *mdx* replicates by each other and excluding ratios larger than 1.5 and its reciprocal (i.e. 95% confidence interval). Secondly, proteins were considered regulated if they had a fold change greater than 1.35 or lower than 0.74 (i.e. 90% confidence interval).

## miRNA and mRNA Microarray Processing and Statistical Analysis

Each RNA sample was analysed using the Agilent 2100 Bioanalyzer (Agilent Technologies, USA) to assess RNA quality. 500 ng of total RNA was run for each of the 12 samples on Affymetrix GeneChip® miRNA 3.0 arrays (Affymetrix, Santa Clara, CA), and 100 ng of column-purified (RNeasy Mini kit, Qiagen) total RNA was run on Mouse Gene ST 1.1 Array plates. Labeling and hybridization were performed according to standard Affymetrix protocols at the Affymetrix Core facility BEA, Bioinformatics and Expression Analysis, at Novum, Huddinge, Sweden. The processing and data analyses were performed in Affymetrix Expression

Console Software.

The miRNA arrays were analyzed using Robust Multichip Analysis (RMA) and DABG (detected above background). Since the miRNA 3.0 array covers 153 organisms we sorted out *Mus musculus* transcripts only. Transcripts with Absent detection signals in at least three of the four replicates (or two out of the three replicates in the controls) were removed. The ST1.1 arrays were analysed using Median polish, RMA background correction and Sketch-Quantile normalization (RMA-Sketch in Expression Console Software). Only the signals from the probe sets in the main category were used. If a probe set signal was below 30 in all the groups, it was removed. The criterion for differentially expressed transcripts was set to *P*<0.05 (unpaired t-test, 2-sided). False Discovery Rate (FDR) estimations were done calculating *q* values (*qvalue* R package) from the *P*-value lists. The microarray data discussed in this study have been deposited in NCBI’s Gene Expression Omnibus (3) and are accessible through GEO Series accession number GSE64420 (<http://www.ncbi.nlm.nih.gov/geo/query/acc.cgi?acc=GSE64420>).

**Exon Skipping RT-qPCR**

To assess levels of *Dmd* exon 23 skipping, quantitative real-time PCR was used. 1 µg of RNA was reverse transcribed using the High Capacity cDNA RT Kit (Applied Biosystems, Warrington, UK) according to manufacturer’s instructions. qPCR analysis was performed using 25 ng cDNA template and amplified with TaqMan Gene Expression Master Mix (Applied Biosystems, Warrington, UK) on a StepOne Plus Thermocycler (Applied Biosystems). Levels of *Dmd* exon 23 skipping was determined by multiplex qPCR of FAM-labelled primers spanning Exon 20-21 (Assay Mm.PT.47.9564450, Integrated DNA Technologies, Leuven, Belgium) and HEX-labelled primers spanning Exon 23-24 (Mm.PT.47.7668824, Integrated DNA Technologies, Leuven, Belgium). Exon skipping efficacy was expressed as the percentage of mature *Dmd* transcripts lacking exon 23 following normalisation of exon 23-24 expression levels to exon 20-21 levels.

**Western Blot**

To assess dystrophin restoration via western blot, 8 µm transverse sections of tissue were lysed in buffer (75 mM Tris–HCl (pH 6.5), 10 % sodium dodecyl sulphate, 5 % 2-mercaptoethanol and protease inhibitors). Proteins were resolved using the NuPAGE Tris-Acetate system (using 3-8% pre-cast gels) in conjunction with the XCell SureLock™ Mini-Cell system (Life Technologies) according to the manufacturer’s protocol. Proteins were transferred to an Immobilon-FL PVDF membrane (Millipore) using the NuPAGE XCell II™ blotting apparatus. Both membrane blocking and antibody incubations were performed in Odyssey Blocking Buffer (LiCOR). The membrane was probed with monoclonal anti-dystrophin (1:200, NCL-DYS1, Novocastra) and anti-vinculin (1:100,000, hVIN-1, Sigma, acting as a loading control) antibodies. Secondary antibody IRDye 800CW goat anti-mouse was used at a dilution of 1:20,000 (LiCOR). After washing fluorescence was detected and quantified using the Odyssey imaging system (LiCOR).

**MicroRNA Target Finder**

The IPA microRNA Target Filter was used to identify possible miRNA regulatory events associated with dystrophic pathology. Only well-established DMD-associated dystromiRs (miR-21, miR-29c, miR-31, miR-34c, miR-146b and miR-206) were considered. DystromiR expression was compared with the matched transcriptomics dataset and filtered to include only discordant expression pairings (i.e. miRNA up, mRNA down or miRNA down, mRNA up). This analysis identified 2,111 miRNA-mRNA interactions (**File S4**). To filter the data, the results were limited to targets with fold changes >1.5, and to only those interactions with experimental evidence or high confidence prediction. After filtering, miRNA-targets schemas were generated and mRNA expression data overlaid (>1.5 fold changes only). Predicted miRNA-protein interactions are listed in **File S5**.

**Supplementary References**

1. Ge,Y., Molloy,M.P., Chamberlain,J.S. and Andrews,P.C. (2003) Proteomic analysis of mdx skeletal muscle: Great reduction of adenylate kinase 1 expression and enzymatic activity. *Proteomics*, **3**, 1895–1903.

2. Doran,P., Dowling,P., Lohan,J., McDonnell,K., Poetsch,S. and Ohlendieck,K. (2004) Subproteomics analysis of Ca+-binding proteins demonstrates decreased calsequestrin expression in dystrophic mouse skeletal muscle. *Eur. J. Biochem.*, **271**, 3943–3952.

3. Ge,Y., Molloy,M.P., Chamberlain,J.S. and Andrews,P.C. (2004) Differential expression of the skeletal muscle proteome in mdx mice at different ages. *Electrophoresis*, **25**, 2576–2585.

4. Doran,P., Dowling,P., Donoghue,P., Buffini,M. and Ohlendieck,K. (2006) Reduced expression of regucalcin in young and aged mdx diaphragm indicates abnormal cytosolic calcium handling in dystrophin-deficient muscle. *Biochim. Biophys. Acta*, **1764**, 773–785.

5. Doran,P., Martin,G., Dowling,P., Jockusch,H. and Ohlendieck,K. (2006) Proteome analysis of the dystrophin-deficient MDX diaphragm reveals a drastic increase in the heat shock protein cvHSP. *Proteomics*, **6**, 4610–4621.

6. Gulston,M.K., Rubtsov,D.V., Atherton,H.J., Clarke,K., Davies,K.E., Lilley,K.S. and Griffin,J.L. (2008) A combined metabolomic and proteomic investigation of the effects of a failure to express dystrophin in the mouse heart. *J. Proteome Res.*, **7**, 2069–2077.

7. Doran,P., Wilton,S.D., Fletcher,S. and Ohlendieck,K. (2009) Proteomic profiling of antisense-induced exon skipping reveals reversal of pathobiochemical abnormalities in dystrophic mdx diaphragm. *Proteomics*, **9**, 671–685.

8. Lewis,C. and Ohlendieck,K. (2010) Proteomic profiling of naturally protected extraocular muscles from the dystrophin-deficient mdx mouse. *Biochem. Biophys. Res. Commun.*, **396**, 1024–1029.

9. Lewis,C., Jockusch,H. and Ohlendieck,K. (2010) Proteomic Profiling of the Dystrophin-Deficient MDX Heart Reveals Drastically Altered Levels of Key Metabolic and Contractile Proteins. *J. Biomed. Biotechnol.*, **2010**, 648501.

10. Gardan-Salmon,D., Dixon,J.M., Lonergan,S.M. and Selsby,J.T. (2011) Proteomic assessment of the acute phase of dystrophin deficiency in mdx mice. *Eur. J. Appl. Physiol.*, **111**, 2763–2773.

11. Guevel,L., Lavoie,J.R., Perez-Iratxeta,C., Rouger,K., Dubreil,L., Feron,M., Talon,S., Brand,M. and Megeney,L.A. (2011) Quantitative proteomic analysis of dystrophic dog muscle. *J. Proteome Res.*, **10**, 2465–2478.

12. Carberry,S., Zweyer,M., Swandulla,D. and Ohlendieck,K. (2012) Proteomics reveals drastic increase of extracellular matrix proteins collagen and dermatopontin in the aged mdx diaphragm model of Duchenne muscular dystrophy. *Int. J. Mol. Med.*, **30**, 229–234.

13. Carberry,S., Zweyer,M., Swandulla,D. and Ohlendieck,K. (2012) Profiling of age-related changes in the tibialis anterior muscle proteome of the mdx mouse model of dystrophinopathy. *J. Biomed. Biotechnol.*, **2012**, 691641.

14. Yoon,J.H., Johnson,E., Xu,R., Martin,L.T., Martin,P.T. and Montanaro,F. (2012) Comparative proteomic profiling of dystroglycan-associated proteins in wild type, mdx, and Galgt2 transgenic mouse skeletal muscle. *J. Proteome Res.*, **11**, 4413–4424.

15. Rayavarapu,S., Coley,W., Cakir,E., Jahnke,V., Takeda,S., Aoki,Y., Gordish-Dressman,H., Jaiswal,J.K., Hoffman,E.P., Brown,K.J., *et al.* (2013) Identification of disease specific pathways using in vivo SILAC proteomics in dystrophin deficient mdx mouse. *Mol. Cell Proteomics*, 10.1074/mcp.M112.023127.

16. Betts,C., Saleh,A.F., Arzumanov,A.A., Hammond,S.M., Godfrey,C., Coursindel,T., Gait,M.J. and Wood,M.J. (2012) Pip6-PMO, A New Generation of Peptide-oligonucleotide Conjugates With Improved Cardiac Exon Skipping Activity for DMD Treatment. *Molecular Therapy — Nucleic Acids*, **1**, e38.

17. Branca,R.M.M., Orre,L.M., Johansson,H.J., Granholm,V., Huss,M., Pérez-Bercoff,Å., Forshed,J., Käll,L. and Lehtiö,J. (2014) HiRIEF LC-MS enables deep proteome coverage and unbiased proteogenomics. *Nat. Methods*, **11**, 59–62.
